# Short association fibres form topographic sheets in the human V1–V2 processing stream

**DOI:** 10.1162/imag_a_00498

**Published:** 2025-03-10

**Authors:** Fakhereh Movahedian Attar, Evgeniya Kirilina, Denis Chaimow, Daniel Haenelt, Christian Schneider, Luke J. Edwards, Kerrin J. Pine, Carsten Jäger, Katja Reimann, Andreas Pohlmann, João Periquito, Tobias Streubel, Robert Trampel, Siawoosh Mohammadi, Thoralf Niendorf, Markus Morawski, Nikolaus Weiskopf

**Affiliations:** Department of Neurophysics, Max Planck Institute for Human Cognitive and Brain Sciences, Leipzig, Germany; Institute for Anatomy I, Medical Faculty & University Hospital Düsseldorf, Heinrich-Heine-University Düsseldorf, Düsseldorf, Germany; Institute of Neuroscience and Medicine (INM-1), Research Centre Jülich, Jülich, Germany; International Max Planck Research School on Neuroscience of Communication: Function, Structure, and Plasticity, Leipzig, Germany; Department of Neurology, Max Planck Institute for Human Cognitive and Brain Sciences, Leipzig, Germany; Faculty of Science, Radboud University, Nijmegen, The Netherlands; Paul Flechsig Institute, Center of Neuropathology and Brain Research, University of Leipzig, Leipzig, Germany; Berlin Ultrahigh Field Facility, Max Delbrück Center for Molecular Medicine in the Helmholtz Association, Berlin, Germany; Department of Systems Neuroscience, University Medical Center Hamburg-Eppendorf, Hamburg, Germany; Department of Neuroradiology, University of Lübeck, Lübeck, Germany; MR Physics group, Max Planck Institute for Human Development, Berlin, Germany; Experimental and Clinical Research Center, a joint cooperation between the Charité Medical Faculty and the Max Delbrück Center for Molecular Medicine in the Helmholtz Association, Berlin, Germany; Felix Bloch Institute for Solid State Physics, Faculty of Physics and Earth System Sciences, Leipzig University, Leipzig, Germany; Wellcome Centre for Human Neuroimaging, Institute of Neurology, University College London, London, United Kingdom

**Keywords:** brain organisation, retinotopy, structure-function relationships, sub-millimetre resolution, tractography, U-fibres

## Abstract

Despite the importance of short association fibres (SAF) for human brain function, their structures remain understudied. It is not known how SAF are organised across the brain, and how consistent their geometries and locations are across individuals. To address this gap, we mapped the precise structures of SAF in the primary (V1) and secondary (V2) visual cortex in a group of participants*in vivo*and a*post mortem*specimen. We assessed the consistency of SAF geometries and their expected structural and functional topography using probabilistic tractography on sub-millimetre-resolution diffusion-weighted MRI combined with functional MRI retinotopic maps*in vivo*. We found that dense SAF connected V1 and V2, forming sheet structures with retinotopic topography and bearing consistent geometries that resembled the local V1–V2 cortical folding.*In vivo*findings were corroborated by the robust and fine-grained*post mortem*reference. Our*in vivo*approach provides important insights into SAF organisation and could be applied to studies across species on cortical and SAF reorganisation and support neuronavigation.

## 1 Introduction

Short association fibres (SAF) are the most abundant, yet under-explored connections in the human white matter connectome (Meynert, 1885;Schüz & Braitenberg, 2002). SAF mainly connect adjacent cortical areas and are located in the superficial white matter (SWM) directly below the cortex.

Because of their location, structure and function of SAF are tightly linked with the overlying cortex. SAF pathways are expected to follow the local patterns of cortical gyrification, which are mainly U-shaped and thus, SAF are also often called U-fibres. However, despite some empirical evidence (Guevara et al., 2020;Román et al., 2022), the precise geometries and organisations of SAF are not characterised across the brain. For example, unlike the long-range white matter fibre pathways (Van Wedeen et al., 2012), it is not known how SAF are organised across the brain. So far, pyramid-shaped SAF have been demonstrated in the frontal and temporal lobes (Shinohara et al., 2020) and SAF mapped in the primary somatosensory cortex (Pron et al., 2021) suggest a sheet structure. Here, we define a SAF sheet in three-dimensions as a thin layer of SAF bundles arranged parallel to one another along the grey/white matter boundary such that layer thickness corresponds to the direction of the grey/white matter normal. Using our definition, the size of the sheet is much smaller along its thickness than along the length and extent of the fibres along the grey/white matter boundary (Supplementary Fig. S1). Establishing models of SAF geometry and organisation across the brain would allow us to investigate SAF structure as a marker for cortical function and development. Further, it is not known whether SAF geometries, alike the long-range pathways, are consistent across individuals in common anatomical locations connecting specific functional areas. This knowledge would enable reliable atlases of SAF and facilitate comparison with functional neuroanatomy.

Until recently, methodological challenges have hindered mapping the SAF comprehensively across the brain, meaning that their structure and function remained understudied. Mapping the SAF has previously been attempted using diffusion weighted imaging (DWI) tractography (Catani et al., 2011;Chauvel et al., 2022;Li et al., 2019;Pron et al., 2021). However, the relatively low spatial resolutions used will only have allowed a subset of the SAF across the brain to be captured. Recent developments in MRI hardware have enabled sub-millimetre spatial resolution DWI (Jones et al., 2018;Liao et al., 2021;Movahedian Attar et al., 2020;Setsompop et al., 2013,^2018^;Shastin et al., 2022;F. Wang et al., 2021), greatly facilitating accurate and robust mapping of SAF using DWI tractography*in vivo*(Song et al., 2014).

Despite the recent technological advances, validating*in vivo*maps of SAF remains challenging because ground-truth information about SAF geometries and distributions across the brain is currently very limited. Previous*in vivo*studies were either not validated (Chauvel et al., 2022;Li et al., 2019;Pron et al., 2021) or only qualitatively validated using, for example,*post mortem*dissection methods (Catani et al., 2011). In our previous work (Movahedian Attar et al., 2020,2024b), we used the retinotopic organisation of human primary (V1), secondary (V2), and tertiary (V3) visual cortex mapped with functional MRI (Engel et al., 1997;Holmes, 1918,^1945^;Inouye, 1909;Sereno et al., 1995) to validate the corresponding maps of SAF connectivity patterns*in vivo*.

Retinotopic maps in the early visual cortex are an example of a special topography in the brain. Topography is a fundamental organisational and coding principle in the human brain (Bellmund et al., 2016;Jbabdi & Johansen-Berg, 2011;Udin & Fawcett, 1988), describing the ordered spatial patterns of information processing and functional organisation in brain regions. In human white matter, connectional topography has been demonstrated*in vivo*in V1–V2 callosal projections (Saenz & Fine, 2010), cerebellar projections (Steele et al., 2017), and projections in the sub-cortical nuclei (Tian et al., 2020) using DWI tractography (Basser et al., 2000;Conturo et al., 1999) and in the optic radiation tract using functional MRI (H. Wang et al., 2023). We used retinotopic maps in the following to explore the detailed anatomies and topography of SAF and their relation to functional neuroanatomy*in vivo*.

We have characterised the geometry and detailed topography of SAF in a sub-region of the human V1–V2 cortex in the representation of the left lower visual field. V1–V2 SAF may bear potential evolutionary significance, for example, with respect to the location of the lunate sulcus in non-human primates (Allen et al., 2006;Armstrong et al., 1991;Falk, 2014). Moreover, the highly reproducible V1–V2 cortical folding geometry (Hinds et al., 2008,^2009^) and border location (Bridge et al., 2005;Sánchez-Panchuelo et al., 2012) with respect to retinotopic organisation provide a suitable testbed for validation of SAF corresponding to the same functional location. Here, the V1–V2 border was localised using functional MRI (fMRI) retinotopic mapping, providing information about each individual’s retinotopic topography. As expected from retinotopic organisation, our findings demonstrated the structural and functional sheet organisation of V1–V2 SAF, which resembled the V1–V2 cortical folding in the explored region*in vivo*, corroborated by a high-fidelity*post mortem*DWI tractography. The established link between SAF structure and cortical function will enable further investigations, prompted by the expected central role of SAF in brain function, development, pathology, and evolutionary trajectories.

## 2 Materials and Methods

**Fig. 1.**
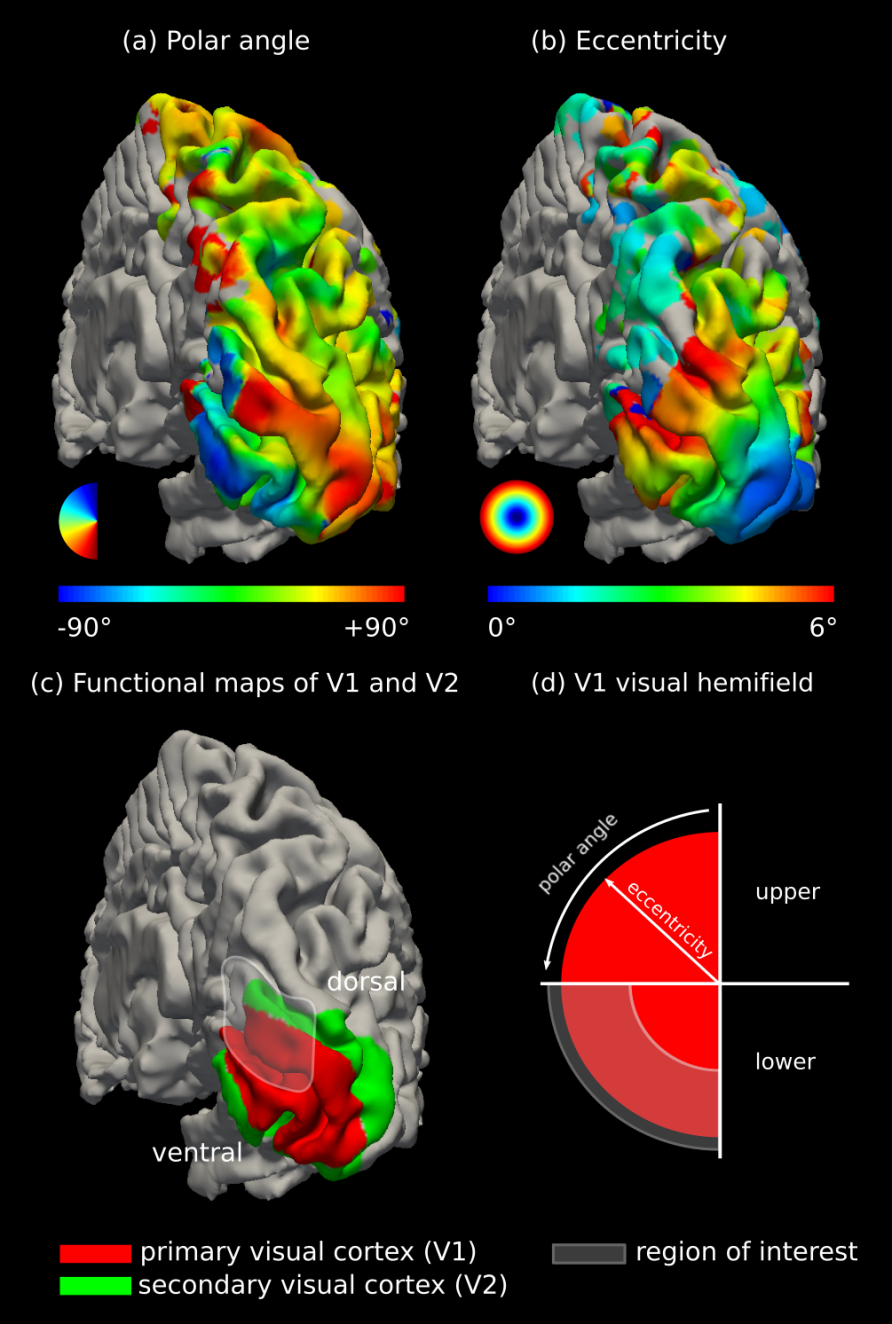
Localisation of the explored V1–V2 cortex*in vivo*using fMRI retinotopic mapping. Smoothed (a) polar angle and (b) eccentricity maps of the right hemisphere of a representative participant. Our fMRI experiment could stimulate the entire visual field in the polar angle and approximately 6.9° in the eccentricity direction. (c) The entire V1–V2 border was manually delineated based on the retinotopic maps. (d) The explored region of interest (ROI) corresponded to a dorsal sub-region in the right hemisphere in the representation of the left lower visual field. The approximate location of the ROI, which was localised using anatomical landmarks, is highlighted in (c) and (d).

**Fig. 2.**
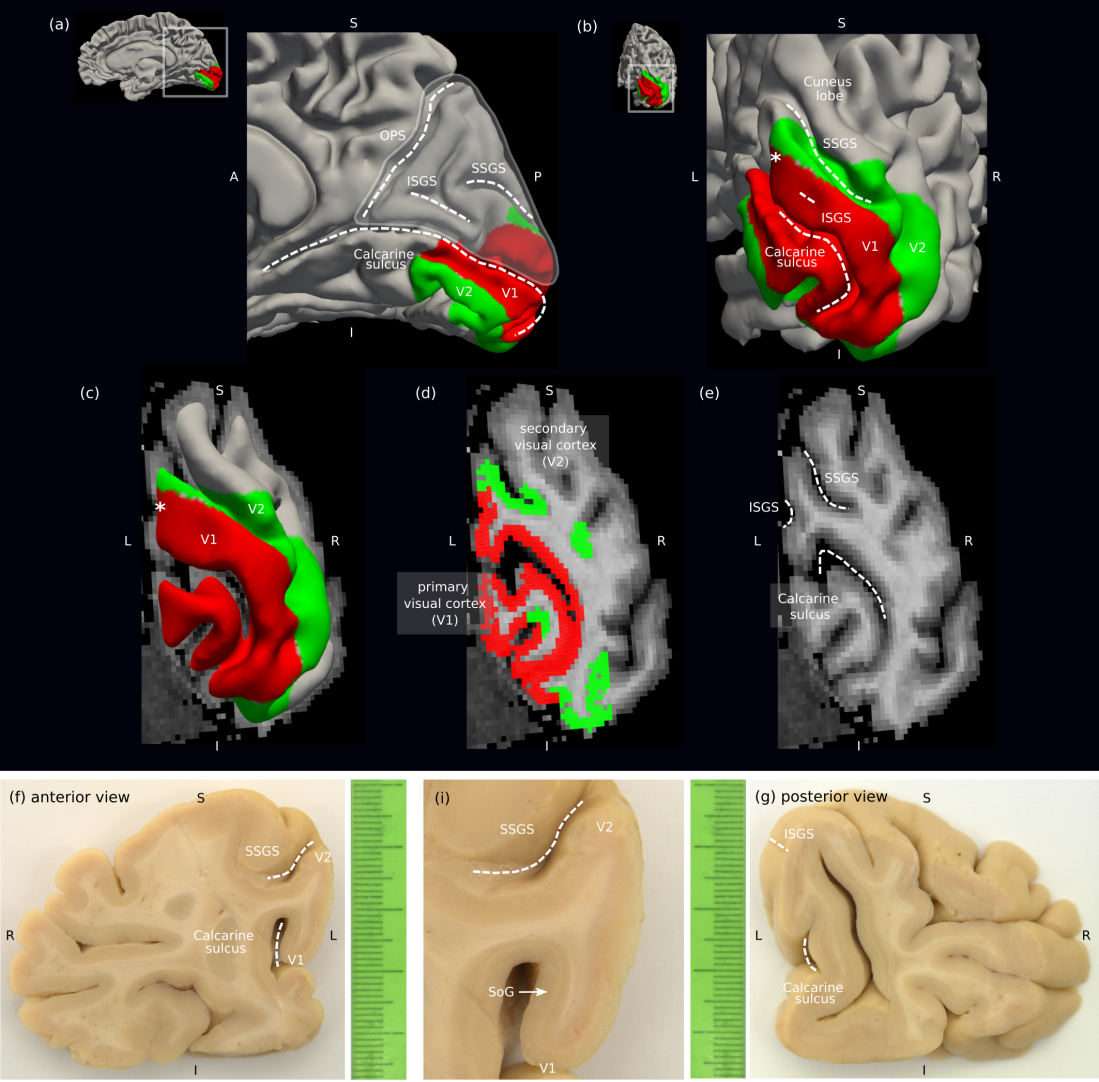
Localisation of the region of interest (ROI) using anatomical landmarks. The ROI was located in a dorsal sub-region in the right hemisphere in the cuneus of the occipital lobe, the latter highlighted in (a). It corresponded to the inferior cuneal gyrus covering the (i) V1–V2 border, mapped using (a–d) functional MRI*in vivo*as shown in a representative participant and (f, g) V1-specific stria of Gennari (SoG) in the*post mortem*specimen. The inferior cuneal gyrus could be localised using (ii) superior sagittal sulcus (SSGS), (iii) inferior sagittal sulcus (ISGS), and (iv) calcarine sulcus (Iaria & Petrides, 2007). These are shown on (a) sagittal and (b) near-coronal surfaces*in vivo*. The gyrus was located above the calcarine sulcus and below the SSGS with the V1–V2 border running almost parallel to the two. The ISGS mapped to the crown of the gyrus and appeared as a shallow sulcus along the ROI in most participants, as shown in (e) a coronal plane in the location of the asterisk (*) in (b, c). (f, g) Localisation of the ROI in the*post-mortem*specimen using the same landmarks. A: anterior, P: posterior, I: inferior, S: superior, L: left, R: right.

We used multi-modal MRI to map and validate the geometry and detailed topography of SAF connecting a dorsal sub-region covering the interface of the human primary (V1) and secondary (V2) visual cortical areas located within the inferior cuneal gyrus (Koutsarnakis et al., 2021) in the right hemisphere (Fig. 1c,Fig. 2). Probabilistic DWI tractography mapped the SAF in the region of interest (ROI), and retinotopic maps from fMRI (Fig. 1;Supplementary Fig. S5) were projected onto the SAF pathways to qualitatively assess their topography in a group of healthy adult participants. Ultra-high spatial resolution probabilistic DWI tractography on*post mortem*human brain tissue was used to corroborate the*in vivo*findings.

Our*in vivo*data were acquired using a multi-modal MRI experimental setup as part of a previous study of SAF in the human brain (Movahedian Attar et al., 2020). Here, we only briefly present the*in vivo*data acquisition, processing, and analysis by highlighting the aspects most relevant to the present study, as well as any modifications to the protocol presented inMovahedian Attar et al. (2020). We present the*post mortem*methods in detail.

### 2.1 *In vivo* imaging

#### 2.1.1 Subject information and data acquisition

Data of 17 healthy participants (9 female and 8 male; age 26 ± 3 years) fromMovahedian Attar et al. (2020)were included. DWI (isotropic voxel size 0.8 mm, b-values 0, 800 and 1800 s/mm^2^, and 60 diffusion-encoding directions per diffusion-weighted shell) were acquired on a 3 T Connectom MRI scanner (Siemens Healthcare, maximum applied gradient amplitude 188 mT/m) using a 32-channel whole-brain coverage radio-frequency receive coil. On a 7 T MRI scanner (Siemens Healthcare), fMRI were acquired to obtain maps of V1 and V2 using retinotopic mapping (Engel et al., 1997;Sereno et al., 1995) and structural MRI were acquired to reconstruct cortical surfaces with FreeSurfer (Fischl, 2012).

Visual stimulation in fMRI retinotopic mapping consisted of a phase-encoded paradigm (Engel et al., 1997;Sereno et al., 1995) with a contrast-reversing black-and-white checkerboard stimulus. Stimulation was restricted to a clockwise/anticlockwise rotating ray along the polar angle (Fig. 1a) and expanding/contracting rings along the eccentricity (Fig. 1b) directions. Four runs of the experiment (2 polar angle, 2 eccentricity) were acquired during which participants were asked to maintain their gaze at a central fixation cross without any attention fixation task (Movahedian Attar et al., 2020). The visual field sign maps used to delineate the V1 and V2 borders were computed based on the phase responses at the stimulus frequency.

#### 2.1.2 Smoothing the retinotopic maps

In our updated protocol, the eccentricity and polar angle maps used to determine SAF topography*in vivo*were first smoothed on the reconstructed cortical surfaces (FreeSurfer v6.0.0) (Fig. 1a, b) to mitigate the effects of acquisition noise. First, surface vertices with reliable functional signal were identified. These showed significant signal deviation from the baseline fMRI signal, determined using an arbitrarily defined signal-to-noise ratio (SNR) threshold of 5. SNR was computed as the magnitude of the signal at the stimulus frequency divided by the standard deviation of the frequency spectrum of the entire fMRI time-series. Functional data points with SNR below the threshold were unreliable and, thus, excluded. Second, for a local neighbourhood (Hagler et al., 2006) around each remaining data point containing only reliable functional signal, the mean retinotopy was computed. The process was repeated 4 times to achieve sufficient smoothing.

#### 2.1.3 Mapping V1 and V2 labels

V1 and V2 borders were manually delineated along the meridians of the visual field corresponding to the functionally defined areal borders of V1 and V2 using the smoothed fMRI retinotopic maps on inflated cortical surfaces. We made sure not to introduce any spatial overlaps between the V1 and V2 labels. The labels were subsequently transformed to DWI volume space using non-linear registration, which introduced some overlap between V1 and V2 because of the interpolation step, affecting the border regions and tight junctions adjoining them. To avoid detecting false-positive SAF pathways, we eliminated the overlaps by assigning the affected voxels to either V1 or V2 based on a probability map.

#### 2.1.4 Localisation of the V1–V2 region of interest

Common anatomical landmarks were used to localise the ROI covering a dorsal sub-region of V1–V2 in the inferior cuneal gyrus (Koutsarnakis et al., 2021) in the right hemisphere, making sure it was consistently placed in the same anatomical location in the*in vivo*participants and the*post mortem*tissue sample to facilitate validation. The following landmarks were used (Fig. 2a–e): (i) The border interfacing the V1 and V2 cortex, to map the SAF connecting them. FMRI retinotopic mapping localised the functional border between V1 and V2 throughout the V1 and V2 cortex*in vivo*. (ii) Calcarine sulcus, (iii) superior sagittal sulcus (SSGS), and (iv) inferior sagittal sulcus (ISGS) (Iaria & Petrides, 2007) were then used to localise the ROI within the explored V1–V2 sub-region.

The calcarine sulcus, which defines the inferior border of the ROI, was used to localise the portion of V1 located directly above it in the inferior cuneal gyrus. The calcarine sulcus is located on the medial occipital lobe and extends from immediately below the splenium of the corpus callosum where it meets the occipito-parietal sulcus (OPS) all the way to the occipital pole along the anterior-posterior axis (Fig. 2a, b). The SSGS, which defines the superior border of the ROI, is located immediately dorsal to the ISGS and was used to localise the portion of V2 located directly below it (Fig. 2a, b). The ISGS is located immediately dorsal to the main body of the calcarine sulcus, running almost parallel to it, and immediately ventral to the SSGS, encapsulated by the two sulci (Fig. 2a, b). The ISGS corresponds to a shallow sulcus on the crown of the V1–V2 gyrus and its appearance was used to determine the extent of the ROI in the anterior-posterior direction. Anatomical MRI (0.7 mm isotropic resolution) and their cortical surface reconstructions were used to locate the sulci. The ISGS could be detected for all participants though not at all locations and with differing shapes, sizes, and distributions in the anterior-posterior direction (Supplementary Fig. S5). The posterior border of the ROI was placed slightly posterior to the detected ISGS to account for potential errors in surface reconstruction, resulting in these shallow sulci appearing flat (seeSupplementary Fig. S2). Given that cortical geometry is rather reproducible in the V1–V2 cortex (Ribeiro et al., 2021), the ROI represented roughly the same functional location across individuals.

We estimated the approximate location of the ROI*in vivo*based on its distance from the occipital pole in the anterior-posterior direction from a representative near-coronal plane located at the posterior end of the ISGS or a location immediately posterior to it. The plane was adjusted so that it would be approximately parallel to the trajectories of the mapped V1–V2 SAF within the ROI.

Our fMRI experiment could map V1 and V2 cortical areas corresponding to only up to an eccentricity of 6.9°, thus restricting SAF mapping to within this region. To enable SAF mapping beyond the mapped V1 and V2 cortex for better characterisation of their geometries, we extended the V1 and V2 borders manually along the respective meridians of the visual field and the sulcal landmarks (Iaria & Petrides, 2007). We did so in individuals where the sulcal landmarks could be visually localised on the reconstructed curvature maps (9 out of 17 participants; seeSupplementary Fig. S2) beyond the functionally mapped V1 and V2 cortical areas. The sulcal landmarks also helped to localise the ROI for participants where fMRI quality was affected by low SNR (3 out of 17 participants affected; seeSupplementary Fig. S2).

#### 2.1.5 DWI fibre modelling and tractography

A crossing fibre and partial volume effect model was used to address the complex fibre arrangements in the superficial white matter. We used the unsupervised implementation of the multi-shell multi-tissue constrained spherical deconvolution (MSMT-CSD) approach (Dhollander et al., 2016;Jeurissen et al., 2013;Tournier et al., 2004) in MRtrix3 (v3.0.0) (Tournier et al., 2019) to map the fibre orientation distribution functions (ODF).

A probabilistic streamline tractography algorithm was used as implemented in MRtrix3 (v3.0.0) (Tournier et al., 2010,^2019^). We updated our original tractography (Movahedian Attar et al., 2020) to use cortical instead of whole-brain seeding. We used the V1 and V2 labels in DWI space as seed masks for tracking, which enabled a high seed resolution of 4 × 4 × 4 per voxel and additionally significantly reduced the output tractogram size compared to the whole-brain approach. The tracking parameters were the same: curvature threshold of 30°, fibre ODF amplitude threshold of 0.1, tracking step size of 0.2 mm, and reconstructed streamline lengths 3–120 mm.

#### 2.1.6 Mapping the V1–V2 SAF pathways

First, SAF connecting the entire V1 and V2 cortex were mapped as streamlines that traversed both the V1 and V2 labels. Second, SAF connecting V1–V2 within the localised ROI were filtered using manually defined 3D inclusion masks covering the ROI for each participant, separately. The mask covered the superficial as well as areas in the deep white matter to avoid removal of SAF streamlines. If necessary, a manually defined exclusion mask was used to remove the streamlines that exited the superficial white matter, belonging to the long-range pathways. The mask was defined in a region located at a depth greater than roughly 2 mm below the cortex estimated at sulcal fundi. For a few affected participants, it also covered near the occipital pole where the tight and tortuous junctions, determined visually, could result in spurious tractography pathways.

SAF geometries were characterised for each participant by their average (mean ± standard deviation) lengths and bending angles computed over all the streamlines using an in-house developed MATLAB script. The bending angle was computed for each streamline as the angle subtending the two 3D vectors that connected each of the two endpoints of the streamline to the point of highest curvature (zero second derivative) along the streamline (Supplementary Fig. S11). It was assumed that the point of highest curvature corresponded roughly to the fundus of the sulcus overlying the SAF bundle. Smaller bending angles corresponded to more U-shaped and larger bending angles corresponded to more V-shaped SAF.

The expected sheet structure of V1–V2 SAF mapped in the ROI was characterised by size across three dimensions, where size was computed as the variance of SAF streamline coordinates along each dimension. For SAF with V-shaped geometries characteristic to small overlying sulcal curvatures, size is much smaller along the sheet thickness than along its length (fibre length) and extent (along the grey/white matter boundary). Recall that we defined a sheet as a thin layer of fibre bundles arranged parallel to one another, extending along the grey/white matter boundary with layer thickness corresponding to the smallest dimension of the sheet. Compared to a sheet, a bundle would exhibit much larger size along the length of the fibres and much smaller and comparable sizes along the two dimensions defining its cross-section (seeSupplementary Fig. S1). To visualise the sheet structure, we fit a 2D surface to SAF streamline coordinates along the largest two dimensions of the sheet and determined the thickness of the surface as size along the smallest dimension of the sheet, for a representative participant. The average (mean ± standard deviation) size along each dimension is reported over all participants.

#### 2.1.7 Combining V1–V2 SAF with retinotopic maps

To visually assess the retinotopic topography of the mapped V1–V2 SAF, we projected the smoothed retinotopic maps onto the SAF streamlines. First, SAF streamlines were transformed from DWI to surface space using a deformation field computed based on a non-linear registration between DWI and structural MRI in ANTs (Avants et al., 2009). Second, retinotopic maps projected onto each vertex on the reconstructed white surface mesh, which marked the grey/white matter boundary, were assigned to the V1–V2 streamlines. To project these values onto each streamline, we identified the three vertices surrounding the streamline’s endpoints in V1 and V2 on the surface mesh using an in-house developed python script (https://github.com/dchaimow/streamline_positions_from_surfaces/tree/v1.0.0), taking them as the fibre’s true cortical terminations. Mean eccentricity and polar angle were computed for each endpoint based on values on the three surrounding vertices, giving four retinotopy values per streamline. Here, we projected the estimated mean retinotopy values on the V1 end of the V1–V2 streamlines onto their entire lengths. To avoid bias from streamlines running entirely within the cortex, we removed streamlines with more than 80 % intra-cortical length.

### 2.2 *Post mortem* imaging

#### 2.2.1 Tissue preparation

The*post mortem*human brain tissue block was provided by the former Brain Banking Centre Leipzig of the German Brain-Net operated by the Paul Flechsig Institute (PFI) Centre of Neuropathology and Brain Research, Medical Faculty, University of Leipzig. The entire procedure of case recruitment, acquisition of the patient’s personal data, protocols, and informed consent forms as well as performing the autopsy and handling the autopsy material were approved by the responsible authorities (Approvals # 282-02 and # 205/17-ek).

The tissue block was obtained from the occipital lobe of the right hemisphere of a 60 year-old male donor who died due to acute respiratory distress syndrome, without known neurological abnormalities. The size of the*post mortem*tissue block was approximately 40 mm × 40 mm × 7 mm. The smallest dimension of the tissue block corresponded to the anterior-posterior direction (Fig. 2) and determined the extent of the ROI, and therefore of the mapped SAF*post mortem*. The*post mortem*interval prior to fixation was less than 48 h, and the tissue block was fixed based on the procedures of the PFI. It was first immersion-fixed in a 4 % paraformaldehyde (PFA) solution in phosphate-buffered saline (PBS) with pH = 7.4 for a duration of at least 6 weeks. The tissue was then washed in a solution of PBS and 0.1 % sodium azide to remove the PFA over 3 consecutive days prior to the first MRI examination.

The tissue block was too large to fit into the radio-frequency coil used for imaging in the small bore MRI system. Therefore, a specimen covering the V1 and V2 interface on the crown of the gyrus in the inferior cuneal gyrus was dissected from the original tissue block, consistent with the location of the V1–V2 ROI*in vivo*(Fig. 2f–g) and the Stria of Gennari (SoG)*post mortem*. Similar to*in vivo*, the V1–V2 gyrus in the specimen was located above the calcarine sulcus and below the superior sagittal sulcus (SSGS). The V1–V2 border in the specimen corresponded roughly to the posterior end of the localised inferior sagittal sulcus (ISGS), indicated by the V1-specific SoG (Fig. 2f, g). This meant that the V1–V2 gyrus corresponded functionally to the representation of the left lower visual field. The size of the dissected specimen was approximately 20 mm × 20 mm × 7 mm and it was located approximately 2.5 cm from the occipital pole, measured along the brain’s anterior-posterior axis and corresponding roughly to the location of the asterisk (*) inFigure 2b. For scanning, the specimen was embedded in the proton-free, MR-invisible fluid Fomblin (Solvay Solexis, New Jersey, USA) and placed in a cylinder-shaped 20 mL syringe (B. Braun SE, Melsungen, Germany) with outer diameter 20 mm.

#### 2.2.2 DWI acquisition

*Post mortem*DWI were acquired on a small-bore ultrahigh field 9.4 T animal scanner (BioSpec 94/20, Bruker, Ettlingen, Germany) tailored to small sample imaging. A 2-channel transceiver MRI CryoProbe operating in quadrature mode was used, designed for high-sensitivity small sample imaging. To ensure high SNR in the V1–V2 gyrus and its adjacent superficial white matter, the V1–V2 cortex was positioned to face the coil surface.

A 2D segmented spin echo EPI sequence was used to acquire the*post mortem*DWI. A small voxel size (0.2 mm × 0.2 mm × 0.225 mm) was used for high-quality imaging of the thin superficial white matter. Multi-shell DWI (b = 1000, 4000, 8000, 12000, 16000 s/mm^2^) were acquired to model the crossing fibre and partial volume effects. DWI shells were acquired in an interleaved order each along 60 non-collinear diffusion encoding directions distributed uniformly on the half sphere (Jones et al., 1999). The imaging parameters were: echo time (TE) = 29.5 ms, volume repetition time (TR) = 18 s, excitation flip angle = 90°, partial Fourier = 3/5, phase encoding steps per segment = 60, acquisition of 4 k-space segments with 15 phase-encoding lines, giving a 100 × 60 matrix which was zero-filled (partial-Fourier) to a matrix size of 100 × 100 before reconstruction, number of slices = 50 positioned parallel to the coil surface. The acquisition was repeated 9 times to enhance SNR. A reference non-diffusion weighted image (*b*= 0 s/mm^2^) was acquired prior to each repetition. Total acquisition time was approximately 54 h.

#### 2.2.3 DWI preprocessing

The DWI were preprocessed as follows. We registered the DWI from all 9 repetitions of the experiment to compensate for drifts in the main magnetic field of the MRI scanner. We used rigid-body registration implemented in ANTs (Avants et al., 2009). All*b*= 0 s/mm^2^images were registered to the*b*= 0 s/mm^2^image from the second repetition, which resulted in better registration, giving a set of transforms between the repetitions. DWI from each repetition were then registered to the*b*= 0 s/mm^2^image from the same repetition, giving a set of transforms within the repetitions. Finally, these two sets of transforms were combined to give a composite rigid-body transform for each image, which was used to align each image to the*b*= 0 s/mm^2^from the second repetition in a single interpolation step. We included the 7 best co-aligned DWI repetitions, omitting the 2 for which the*b*= 0 s/mm^2^could not be perfectly aligned.

The*b*= 0 s/mm^2^images were affected by localised systematic artifacts in V2 (seeSupplementary Fig. S6). Because of this, we removed the*b*= 0 s/mm^2^images after the registration step, before continuing with subsequent processing and analysis. For the processing steps requiring a*b*= 0 s/mm^2^, we used a pseudo-*b*= 0 s/mm^2^image computed by the spherical mean of all of the*b*= 1000 s/mm^2^images. This was justified by the low diffusivity of fixed*post mortem*tissue (Roebroeck et al., 2019).

#### 2.2.4 DWI fibre modelling and tractography

Similar to*in vivo*, we mapped the fibre distributions using the unsupervised MSMT-CSD approach (Dhollander et al., 2016;Jeurissen et al., 2013;Tournier et al., 2004,^2007^) implemented in MRtrix3 (v3.0.0) (Tournier et al., 2019). The cerebrospinal fluid, which is modelled as a compartment with isotropic diffusion, was not present in the*post mortem*specimen. Therefore, the unsupervised algorithm segmented the tissue into one anisotropic compartment corresponding to the white matter and two isotropic compartments visually corresponding to the upper and lower cortical grey matter layers (Movahedian Attar et al., 2023).

We estimated the thickness of the superficial white matter layer based on the fibre distribution map. We visually identified the number of voxels containing superficial white matter fibres below the cortical grey/white matter boundary, arranged parallel to it, as a measure of its layer thickness. We used a few representative coronal planes and reported the thickness in millimetres.

For visualisation, we computed the diffusion tensor using the weighted linear least-squares approach implemented in MRtrix3 (v3.0.0) (Tournier et al., 2019) based on the*b*= 1000 and*b*= 4000 s/mm^2^DWI, giving maps of fractional anisotropy (FA) and mean diffusivity (MD).

The tractography approach and parameters*post mortem*were the same as*in vivo*. However, the entire sample instead of only the cortex was used as the seed mask, step size was 0.1 mm, optimised for the*post mortem*spatial resolution based on recommendations inTournier et al. (2012), and streamline lengths were restricted to 3–30 mm.

#### 2.2.5 Mapping the V1–V2 SAF pathways

SAF connecting V1 and V2 were mapped using a pair of manually created V1 and V2 cortical masks. We used the V1-specific stria of Gennari visible on the MD maps (Fig. 7b) to create the V1 mask. The V2 mask was the cortical region not identified as V1. Similar to the*in vivo*case, streamlines that traversed both V1 and V2 were included and their geometries were characterised using a length and bending angle metric (Supplementary Fig. S11). The entire V1–V2 gyrus covered in the tissue sample, corresponding to a sub-region of the ROI in the anterior-posterior direction*in vivo*, was used for SAF mapping.

#### 2.2.6 Mapping the topographic order of the V1–V2 SAF

The ordered spatial arrangement, or topography, of SAF connecting V1 and V2 was mapped as a reference for*in vivo*validation. To map the topography*post mortem*, where no functional information is available, we clustered the SAF streamlines based on two separate sets of manually defined labels, which were carefully drawn directly below the cortical grey matter border of V1 (Fig. 2;Supplementary Fig. S16a, c). The first and second sets of labels consisted of 15 and 6 non-overlapping labels in the expected anatomical directions of polar angle and eccentricity, respectively. Using the same initial set of streamlines, the labels allowed us to cluster the SAF based on the locations of their terminations in V1. A unique colour was assigned to the streamlines in each cluster to visualise the topographic order.

## 3 Results

### 3.1 Consistent localisation of functional and gross anatomical V1–V2 interface

**Fig. 3.**
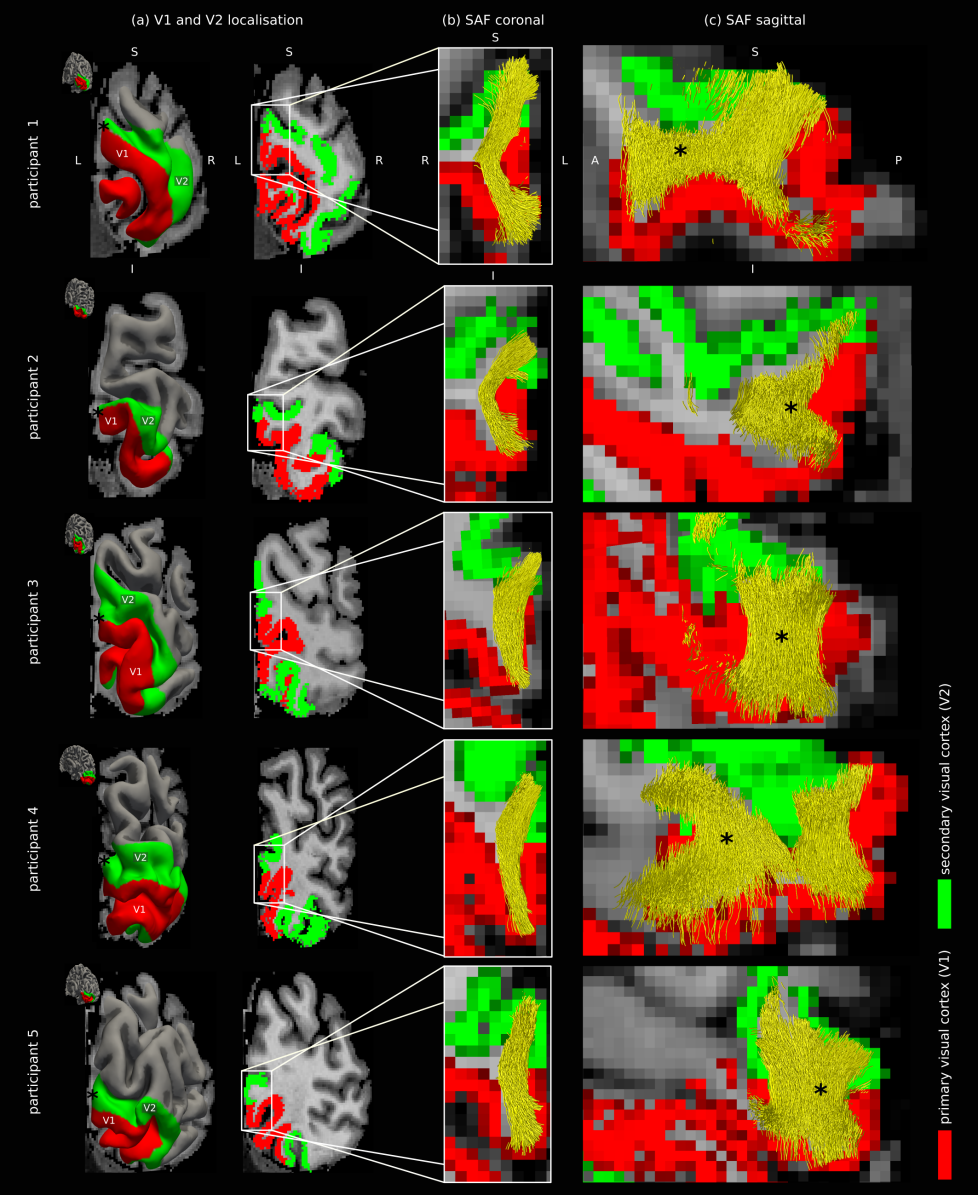
Geometries of the SAF mapped in the localised V1–V2 gyrus*in vivo*were consistent across participants, characterised by a large bending angle. Here, SAF are shown for five representative participants (rows) (See results for other participants inSupplementary Figs. S7, S8 and S9). (a) Surface (left) and volume (right) views of a representative coronal plane along the gyrus, looking from the posterior end. In diffusion space, (b) the mirror view of SAF is shown looking from the anterior end, corresponding roughly to the location of the plane in (a), marked by the asterisk (*) in (a, c), and (c) SAF mapped throughout the gyrus, pointing to a sheet geometry. The corresponding fibre distribution maps and sulcal landmarks are shown inSupplementary Figure S10. A: anterior, P: posterior, I: inferior, S: superior, L: left, R: right.

Using a combination of functional maps of V1 and V2 and sulcal landmarks (Fig. 2a–e), we were able to reliably localise the V1–V2 ROI in the inferior cuneal gyrus for all participants despite the variability in cortical gyrification patterns. The localised ROI and the anatomical landmarks are shown for all participants (9 female, 8 male; age 26 ± 3 years (mean ± standard deviation)) inSupplementary Figures S2, S3 and S4. The localised ROI covered the gyrus at the V1–V2 interface and extended from the cortical representation of, on average, approximately 3° to the experimentally mapped maximum eccentricity of 6.9° (or slightly beyond it; seeSupplementary Fig. S2). We consistently detected a dense band of SAF connecting the V1–V2 gyrus for all participants (Fig. 3;Supplementary Figs. S7, S8 and S9). Proximal to the occipital pole, the highly compact and tortuous cortical gyrification patterns could give rise to spurious tractography streamlines. We excluded the affected areas, detected in 4 out of the 17 participants, from further analyses (see participants 9–12 inSupplementary Fig. S8).

### 3.2 V1–V2 SAF geometries were consistent across participants *in vivo*

The geometries of SAF connecting the V1–V2 gyrus in the ROI were overall highly consistent across participants (Fig. 3). The shapes of the SAF were characterised by a large bending angle of 142 ± 21° (mean ± standard deviation) estimated over the group. The corresponding lengths of the SAF were 12 ± 7 mm (mean ± standard deviation), in line with previously reported values across the human brain (Schüz & Braitenberg, 2002). The large bending angles made the SAF resemble V-shapes (Supplementary Fig. S11). Example SAF geometries are shown on coronal or near-coronal planes along the V1–V2 border in locations either immediately posterior to or directly on the posterior end of the localised inferior sagittal sulcus (ISGS) for all participants (Fig. 3b;Supplementary Figs. S7b, S8b and S9b). Despite their common shapes, the detailed geometries of the SAF varied between participants and also locally along the V1–V2 border for each participant, determined by the local cortical gyrifications. For example, SAF in the locations of the asterisk (*) inSupplementary Figure S8cfor participant 12 andSupplementary Figure S9cfor participant 15 in location (i) showed strong U-shaped geometries as opposed to the V-shapes commonly mapped in the ROI.

The geometries of the SAF appeared almost flat in the shallowest and smallest V1–V2 gyri, resulting in the shortest detected SAF (see location (i) for participants 6 and 7 inSupplementary Fig. S10). Although the flat geometry may highlight the impact of low imaging resolution relative to the size of the gyrus in the smaller gyri, it may also reflect the underlying cortical geometry irrespective of imaging parameters. A complementary analysis performed over all participants demonstrated significantly (p<0.05) smaller bending angles (higher curvature) of SAF in cortical locations where a shallow sulcus was mapped on the crown of the V1–V2 gyrus (mean ± standard deviation 139 ± 21°) compared to locations where the cortex appeared almost flat (mean ± standard deviation 152 ± 15°). This finding points to the potential sensitivity of SAF geometries to differences in cortical folding structure.

### 3.3 The V1–V2 SAF formed sheets with retinotopic topography *in vivo*

**Fig. 4.**
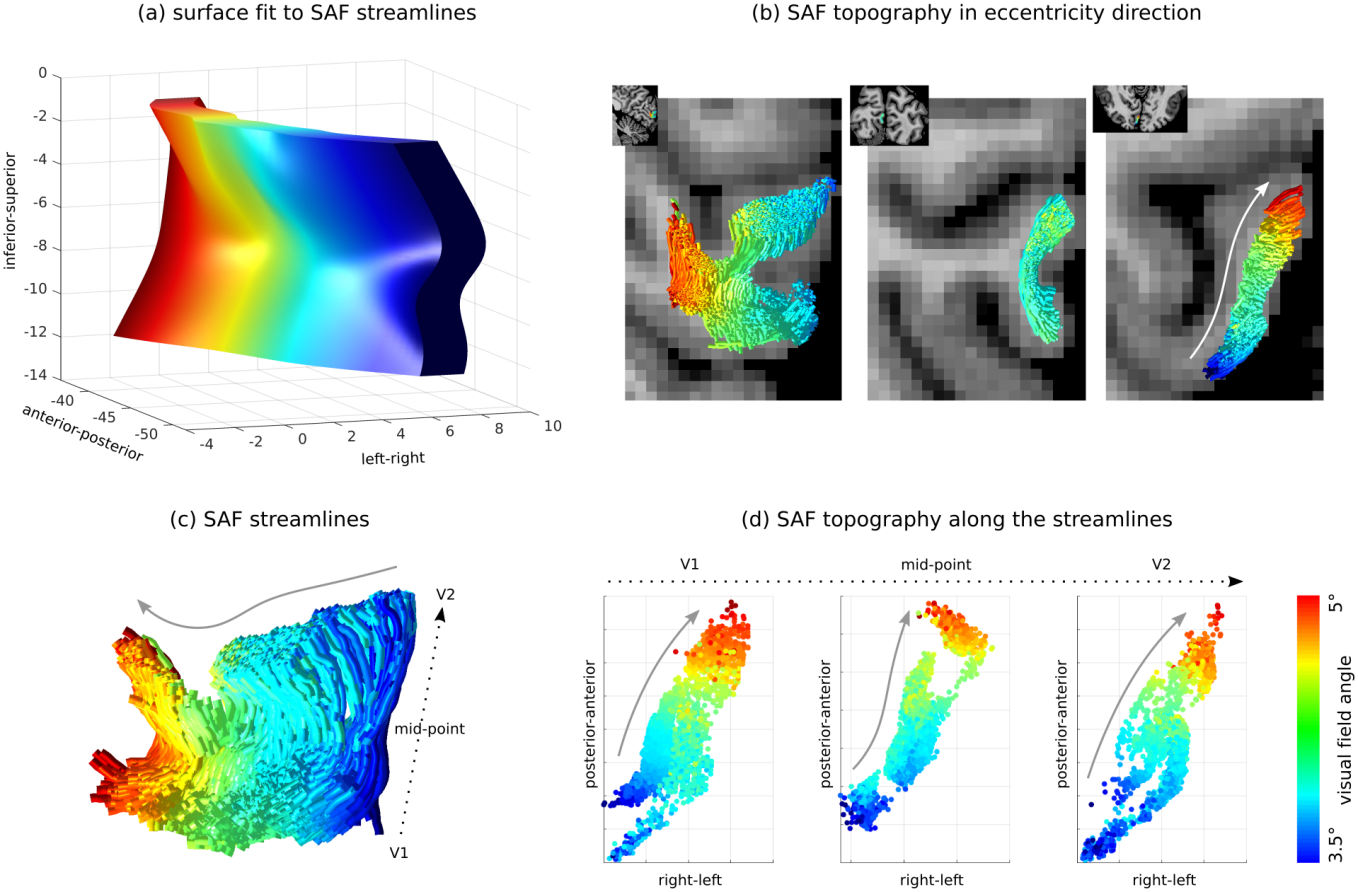
SAF connecting the V1–V2 gyrus exhibited a topographic sheet geometry*in vivo*, demonstrated for a single participant. (a) A 2D surface fit to the SAF streamline dimensions corresponding to the length and extent of the fibres in a representative participant helped to appreciate the sheet geometry. Here, the colour coding represented eccentricity from retinotopic mapping. The surface thickness represented the thickness of the SAF layer, estimated as variance over all streamline coordinates in the respective dimension. (b) Eccentricity projected onto the SAF streamlines confirmed topographic order in the respective anatomical direction (white arrow). (c, d) Topography in the eccentricity direction (grey arrows) is shown at three locations along the SAF streamlines (dotted arrows). For this participant, SAF could be mapped only up to 5° eccentricity (up to 6.9° mapped by fMRI) using tractography possibly because of locally unresolved crossing fibres in the superficial white matter.

The V1–V2 SAF exhibited a sheet geometry*in vivo*(Fig. 4). The first sheet dimension corresponded to the extent of fibres running in the anterior-posterior direction along the V1–V2 border along the calcarine sulcus (grey arrow inFig. 4c) and could be best visualised on sagittal planes (Fig. 3c;Supplementary Figs. S7c, S8c and S9c). The second sheet dimension corresponded to the length of the fibres running within the superficial white matter parallel to the cortical boundary in the inferior-superior direction (dotted arrow inFig. 4c) and could be best visualised on (near) coronal planes (Supplementary Fig. S10). The third sheet dimension, also its smallest dimension, corresponded to the thickness of the SAF layer extending into the white matter from the cortical boundary in the cortical radial direction and could also be best visualised on (near) coronal planes.

For all participants, the computed size was much larger in the first two dimensions of the sheet aligned with the V1–V2 border (roughly in the anterior-posterior direction) and the dominant fibre orientations (perpendicular to the V1–V2 border in the inferior-superior direction), respectively. As expected, we observed the smallest size in the radial direction normal to the cortical surface (corresponding to the thickness of the SAF sheet roughly in the left-right direction), orthogonal to the first two dimensions. The corresponding group-average sizes computed as the variances of streamline coordinates computed over the group were 16 ± 25 mm^2^, 11 ± 6 mm^2^, and 2 ± 1 mm^2^(mean ± standard deviation), respectively. This implies that for shorter V-shaped SAF pathways the variance would be smaller in the second sheet dimension.

**Fig. 5.**
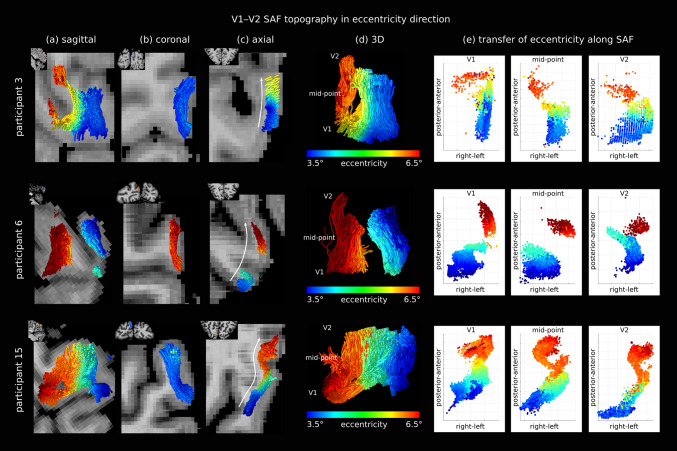
Retinotopic topography was preserved in the V1–V2 SAF sheet in the eccentricity direction*in vivo*. Three representative participants are shown (rows) (See results for other participants inSupplementary Figs. S12 and S13). Eccentricity on the V1 cortex was projected onto each corresponding SAF streamline viewed on (a) sagittal, (b) coronal, and (c) axial slices. Arrows in (c) show the direction of increasing eccentricity, aligned with the extent of the SAF sheet roughly in the anterior-posterior direction. (d) 3D sagittal view of the SAF showing (e) three coordinate locations along the streamlines at which the projected eccentricity is shown to better appreciate along-track topography. See alsoSupplementary Video V.1.

**Fig. 6.**
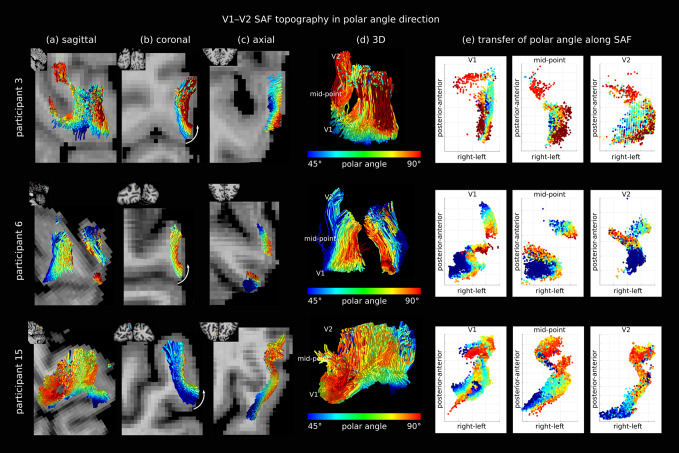
Retinotopic topography was preserved in the V1–V2 SAF sheet also in the polar angle direction*in vivo*but to a lesser extent compared to eccentricity. Three representative participants are shown (rows) (See results for other participants inSupplementary Figs. S14 and S15). Polar angle on the V1 cortex was mapped onto each corresponding SAF streamline viewed on (a) sagittal, (b) coronal, and (c) axial slices. Arrows in (c) show the direction of increasing polar angle, aligned with the thickness of the SAF sheet roughly in the left-right direction. (d) 3D sagittal view of the SAF showing (e) three coordinate locations along the streamlines at which the projected polar angle is shown to better appreciate along-track topography. See alsoSupplementary Video V.2.

The SAF sheet exhibited retinotopic topography*in vivo*(Figs. 4,5, and6;Supplementary Figs. S12, S13, S14, and S15). We explored retinotopy in the SAF sheet qualitatively by visualisation, assessing whether the expected topographic order was preserved in each hemisphere along the SAF pathways going from V1 to V2 in the eccentricity (Fig. 5,Supplementary Figs. S12 and S13) and polar angle (Fig. 6;Supplementary Figs. S14 and S15) directions. We observed that topography was preserved in the eccentricity direction, reflected in the orderly spatial arrangement of the fibres in this direction, demonstrated at three locations along the SAF streamlines (Figs. 4and5;Supplementary Figs. S12 and S13). It was also preserved in the polar angle direction but to a lesser extent, as reflected in the lower spatial order of polar angle values also demonstrated at three points along the SAF streamlines (Fig. 6e;Supplementary Figs. S14e and S15e).

### 3.4 V1–V2 SAF geometries *in vivo* were corroborated by high-fidelity *post mortem* imaging

**Fig. 7.**
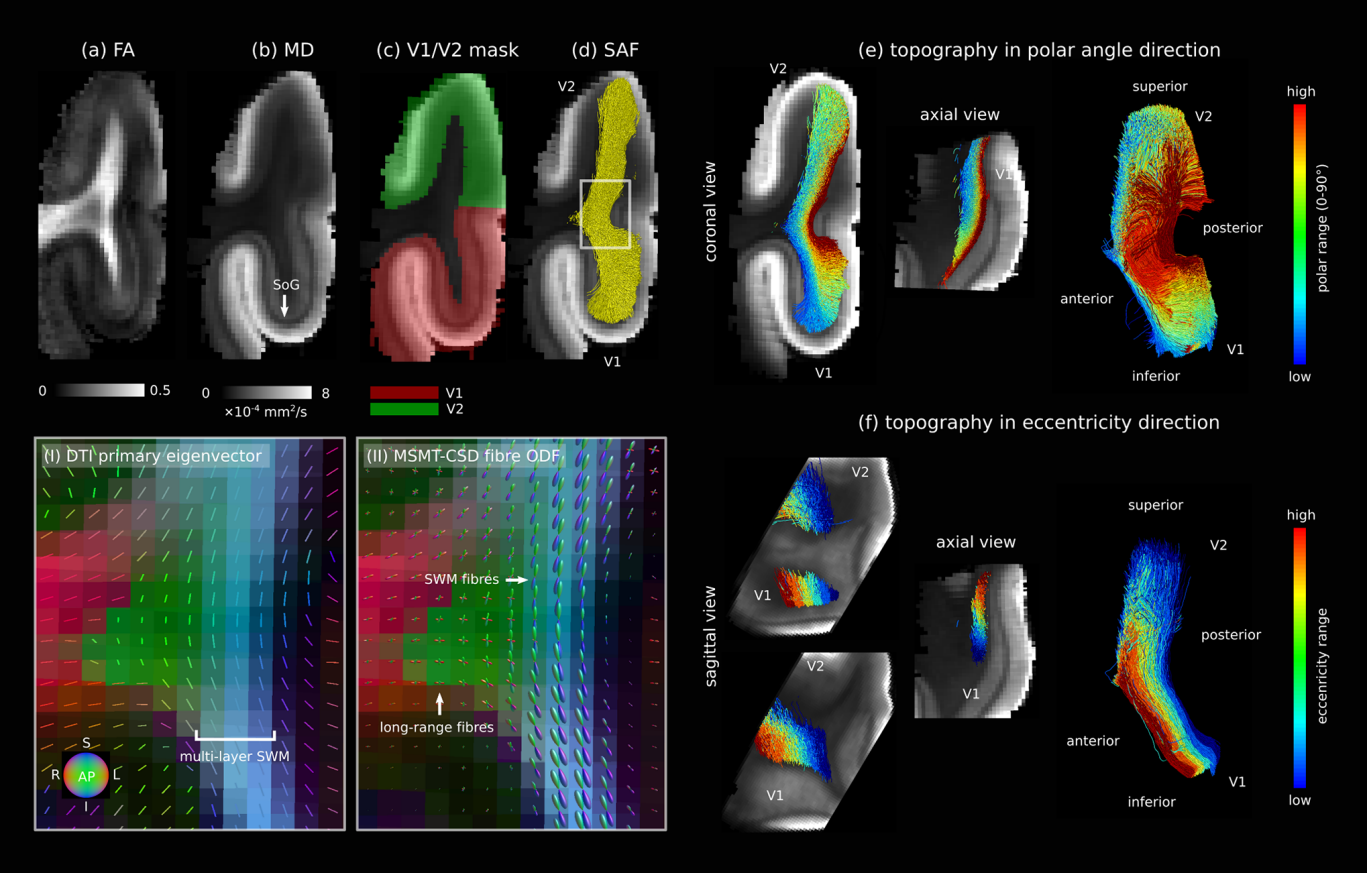
V1–V2 SAF geometry and topography mapped using 0.2 mm isotropic spatial resolution*post mortem*DWI. (a) Fractional anisotropy (FA) and (b) mean diffusivity (MD). Highly myelinated V1-specific Stria of Gennari (SoG) visible on MD. (c) Manually delineated V1 and V2 masks with SoG as landmark were used to map (d) the V1–V2 SAF. For the marked area in (d), dense and multi-layer superficial white matter fibres are shown in (I) DTI primary eigenvector and (II) multi-shell multi-tissue constrained spherical deconvolution (MSMT-CSD) fibre distribution maps. To demonstrate SAF topography, streamlines were clustered based on their location on the V1 cortical border using separate sets of manually created labels in the (e) polar angle and (f) eccentricity directions, giving different sets of output streamlines. V1–V2 SAF were topographically organised along both, as shown in planar and 3D views.

To validate the geometry and topographic order of the V1–V2 SAF mapped*in vivo*, we acquired high-quality*post mortem*DWI of a human specimen obtained from the occipital cortex. The high quality of the*post mortem*DWI was reflected in the highly organised dense and multi-layer structure of the mapped superficial white matter fibres, arranged as ODFs with mainly a single dominant peak parallel to the cortical boundary as expected (Fig. 7;Reveley et al., 2015). Using our*post mortem*DWI, we estimated the thickness of the superficial white matter to be approximately 0.6 mm.

**Fig. 8.**
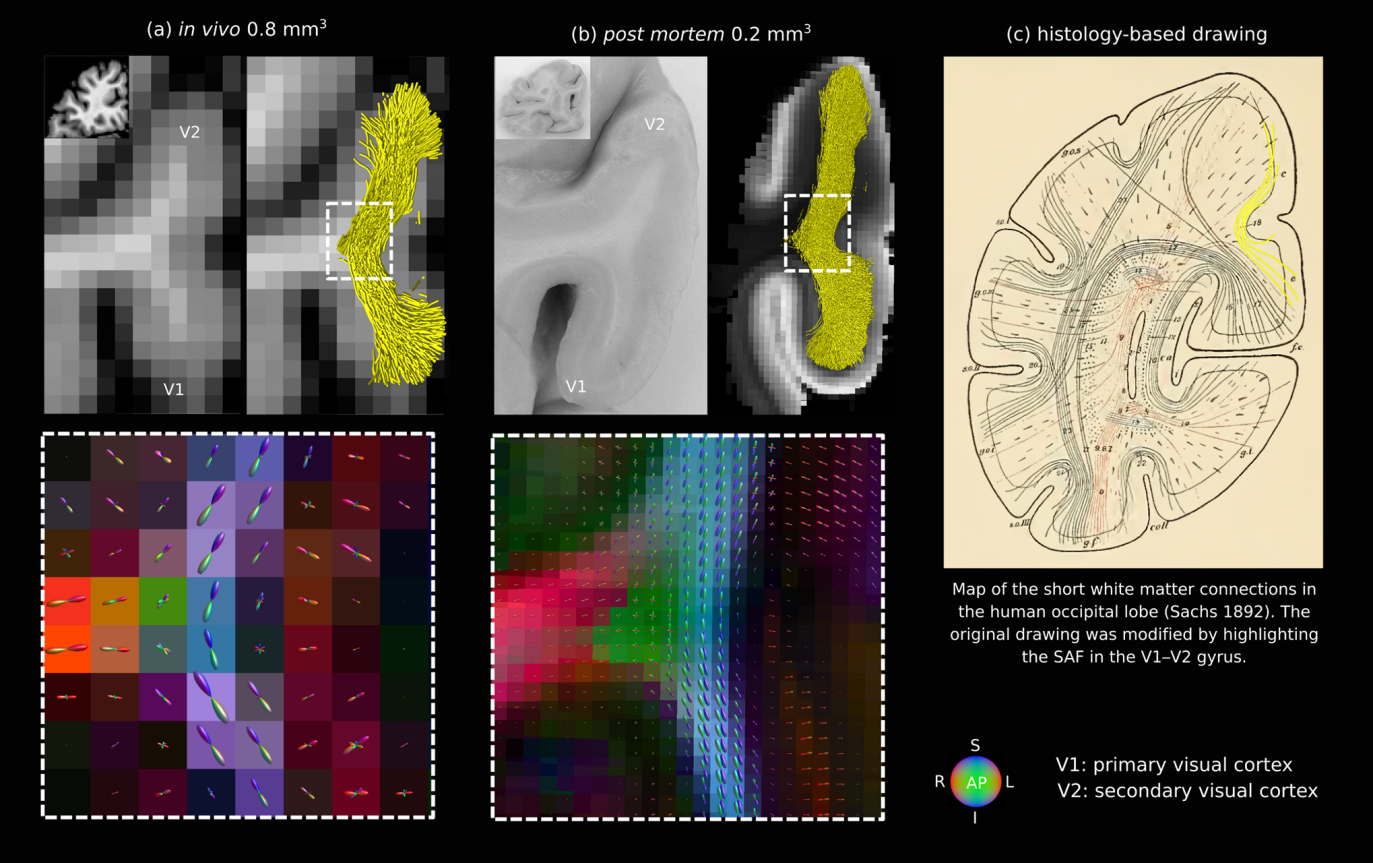
High-quality*post mortem*DWI corroborated the V1–V2 SAF geometries mapped*in vivo*. SAF shown on representative coronal slices in (a) a single participant whose gyral geometry resembled the*post mortem*specimen very closely and (b) the human*post mortem*specimen. The sizes of the V1–V2 gyrus*post mortem*and*in vivo*were 2 cm and 1.4 cm along the inferior-superior axis, respectively, and their approximate distances from the occipital pole were 2.5 cm and 1.8 cm. Fibre orientation maps (dashed-line boxes) were similar in the superficial white matter, showing robustness to increased partial volume effects from adjacent tissue*in vivo*. (c) Historical map of SAF created based on histological fibre mapping (Sachs, 1892) also shows the V1–V2 SAF where the longer SAF were closer to a V-shape and the short U-shaped SAF were closest to the cortical boundary (highlighted in yellow by the current authors).

The geometries of SAF mapped in the V1–V2 gyrus were highly similar*in vivo*and*post mortem*(Fig. 8), assessed based on the length and bending angle metrics. SAF mapped*post mortem*were characterised by a large bending angle of 126 ± 23° (mean ± standard deviation computed over all the V1–V2 streamlines) consistent with the shape of the gyrus. This compared well to the*in vivo*group-average of 142 ± 21°. For a participant whose detailed V1–V2 cortical folding bore high resemblance to the*post mortem*specimen, the mapped SAF geometries also showed striking resemblance (Fig. 8a, b). Quantitatively, for the participant*in vivo*and the*post mortem*specimen the estimated connection lengths were 11 ± 2 mm and 10 ± 3 mm (mean ± standard deviation) and the bending angles were 127 ± 22° and 126 ± 23° (mean ± standard deviation), respectively. Our single-subject*post mortem*observation pointed to the high performance of our*in vivo*approach to map the V1–V2 SAF geometries, which was further corroborated by the reproducibility of the SAF geometries in the group*in vivo*.

The topography of the V1–V2 SAF mapped*in vivo*was also assessed against the*post mortem*reference (Fig. 7e, f). Using two different sets of carefully drawn contiguous labels created on the cortical border of V1 in the expected anatomical directions of eccentricity and polar angle, we clustered the V1–V2 SAF streamlines*post mortem*. Each streamline was assigned a topographic coordinate based on its cluster. We observed that topography was preserved for V1–V2 SAF in both the eccentricity and polar angle directions. Topography appeared to be less well preserved closer to V2 in the polar angle direction (Supplementary Fig. S16), pointing to the general limitations of ultrahigh resolution DWI tractography for mapping the detailed topography of the brain’s anatomical connections even at fine-grained ultrahigh resolutions.

## 4 Discussion

Using high-resolution DWI tractography and fMRI retinotopic mapping*in vivo*, we characterised the precise structures of SAF connecting a sub-region of V1 and V2 in the representation of the left lower visual field in the human brain. We demonstrated a dense band of SAF connecting the V1 and V2 cortex across all individuals in the*in vivo*group. The geometries of the SAF were consistent, showing mainly V-shapes that followed the local gyrification curvature of the cortex along the V1–V2 border throughout the ROI. Importantly, our*in vivo*approach revealed a fundamental structural feature of SAF organisation in these topologically organised cortical areas, the topographic sheet, representing the visual field eccentricity and polar angle coordinates. The*in vivo*findings were corroborated by ultrahigh spatial resolution*post mortem*DWI tractography of a*post mortem*specimen.

It is expected that fibre pathways that share a common origin and destination form bundles or sheets (Dejerine & Dejerine-Klumpke, 1895). However, unlike for the well-studied long-range white matter fibre pathways, the precise geometries of the SAF have not been extensively studied. It is not known whether SAF in common anatomical locations, responsible for common brain functions, also share common geometries and organisations across individuals. We found that SAF in the anatomically-defined ROI in the V1–V2 cortex formed topographically organised sheet structures. This was likely explained by the expected retinotopic organisation of SAF connections in the V1–V2 cortex in both eccentricity and polar angle directions, as this principle requires the SAF to connect all retinotopically corresponding points in V1 and V2, and this would be less efficient if all the streamlines were formed into a bundle (Fig. 4). We also demonstrated consistent shapes of the V1–V2 SAF throughout the ROI in most individuals, in line with the expected highly reproducible V1–V2 cortical folding across individuals.

To confirm our*in vivo*results, we used ultrahigh spatial resolution*post mortem*DWI tractography. The lower resolution of*in vivo*DWI may reduce the sensitivity and introduce bias in our tractography. Using our high-fidelity*post mortem*analysis confirmed the expected dense and highly coherent fibre arrangement in the superficial white matter (Reveley et al., 2015). We observed a strong resemblance between the V1–V2 SAF geometries*in vivo*and*post mortem*, which we quantified using a length and bending angle metric. The estimated lengths were consistent with previously reported values across the human brain (Schüz & Braitenberg, 2002). The V-shaped bending angles were consistent with the implied cortical folding geometry in the interfaces of strongly topographic cortical areas (Rajimehr & Tootell, 2009), corroborating the functional importance of SAF shapes suggested in previous*in vivo*research (Chauvel et al., 2022;Rajimehr & Tootell, 2009). Importantly, striking resemblance in SAF geometry was observed*in vivo*and*post mortem*for an individual whose V1–V2 cortical folding geometry highly resembled the*post mortem*specimen. Also, to facilitate validation, our*in vivo*analysis was restricted to qualitative comparison in a sub-region of the visual field corresponding to approximately 3–6.9° eccentricity. In a related work, we demonstrated the retinotopic topography of SAF quantitatively throughout the mapped V1–V2 cortex (Movahedian Attar et al., 2024a).

Our work has at least three important implications for studies of brain organisation and development.

First, our finding that retinotopic organisation in the V1–V2 cortex was also supported in the underlying SAF implies that our*in vivo*approach can be used to decipher further aspects of brain topography and its relation to function in local networks of cortical and subcortical connectivity. For instance, exploring cortical information transfer via SAF may help to unravel the organisational principles of superficial white matter in supporting brain functions (Jbabdi et al., 2013). Similarly, mapping cortical and SAF topography following injury or as a result of neuropathology may reveal mechanisms of cortical and SAF reorganisation (Baseler et al., 1999) or help to understand the effects of subcortical SAF lesions in pathology such as multiple sclerosis (Miki et al., 1998). Our approach may also be useful in deriving cortical topography from white matter topography in cortical areas where topography is not established or cannot be measured using traditional functional experiments (Knapen, 2021). Further, the detailed knowledge about SAF may inform stimulation techniques such as transcranial magnetic stimulation (TMS), improving their effectiveness and accuracy (Nummenmaa et al., 2014) as well as neuronavigation in neurosurgery applications (Enchev, 2009).

Second, the fundamental principles and mechanisms underlying SAF development and organisation are not yet established. Previous research in the primate brain suggested that long-range white matter pathways were organised along the known principal axes of development and their intersections formed sheet structures (Tax et al., 2016;Van Wedeen et al., 2012). SAF are distinct from the long-range pathways based upon their connectivity range, shape, and location in the brain, as well as origins of development (Yoshino et al., 2020). Therefore, we propose a different definition of a sheet suited to describing the SAF that follow the cortical folding patterns closely. Maps of this sheet structure may help us to understand the fundamental principles of brain connectivity development and organisation in cortical and sub-cortical networks across the brain. For instance, they could be combined with genetic gradients across the brain, shown to influence cortical gyrification development in the early visual cortex (Rajimehr & Tootell, 2009). Further, the demonstrated sheet organisation of SAF in the topologically organised V1–V2 cortex contrasts with their pyramid-shaped organisation in the human frontal and temporal lobes (Shinohara et al., 2020) and agrees with their organisation in the primary sensory-motor system (Pron et al., 2021). Establishing distinct models of SAF organisation may help us to better understand differences in cortical processing and function across the brain and in development.

Third, detailed anatomical maps of the SAF in humans will enhance cross-species comparisons. A DWI tractography study in the chimpanzee brain*in vivo*demonstrated SAF with U-shapes, V-shapes, and other more slightly curved geometries (Chauvel et al., 2022). V-shaped SAF were not detected in the occipital lobe, contrary to our study. Instead, only slightly curved fibres were detected. This observation may be explained by the low DWI spatial resolution (1.9 mm isotropic) used inChauvel et al. (2022)but it may also point to important evolutionary differences in human and non-human primates, also suggested inChauvel et al. (2023). For instance, the existence of the functional homologue of the lunate sulcus, a deep fissure in the occipital lobe of non-human primates (Allen et al., 2006;Armstrong et al., 1991), in humans has been a subject to debate (Falk, 2014). It is thought that evolutionary brain expansion caused a caudal shift in the location of the lunate sulcus and made it only be present in some individuals (Allen et al., 2006;Armstrong et al., 1991). The reliable mapping of SAF as shown in our study may help capturing the brain’s evolutionary structural changes that may help explain its cognitive development and function. Further, the link between SAF bending angle and cortical folding geometry motivates investigations into the relationship between white matter fibre and cortical folding geometries inspired by the hypothesis put forward byVan Essen (1997).

### 4.1 Limitations

We confirmed the geometries of V1–V2 SAF mapped*in vivo*using a single*post mortem*specimen in the corresponding anatomical location. Despite this limitation, we are confident that our*post mortem*study was conclusive because the SAF geometries were also consistent across individuals in the*in vivo*group. Further validation of the*in vivo*approach may consider SAF in other parts of the brain where smaller cortical geometries are subject to increased partial volume effects or high cortical gyrification angles (U-shaped SAF;Supplementary Fig. S11) give rise to complex fibre arrangements that may be more difficult to resolve.

Our study inherits the general limitations of fMRI retinotopy and DWI tractography.

Only up to approximately 6.9° of the visual field could be stimulated in the eccentricity direction using our fMRI experimental setup. This resulted in limited coverage of the V1–V2 cortex and so the extent of the corresponding SAF mapped*in vivo*. We manually extended the mapped V1–V2 cortex slightly beyond this region by carefully projecting the V1–V2 borders along the sulcal landmarks and visual field meridians in the eccentricity direction. Recent developments in deep learning approaches may be considered to map eccentricity beyond the functionally stimulated cortex (Ribeiro et al., 2021).

The observed SAF topography was less ordered in the polar angle compared to the eccentricity direction, potentially because of the tight arrangement of the fibres in the superficial white matter in the former, especially near the sulcus. Such complex fibre arrangements pose a challenge to tractography, especially at the single-subject level. This finding was based on visual investigation and lacked quantification. In a different work, we quantitatively demonstrated the poorer performance of our method in the polar angle direction using the same dataset (Movahedian Attar et al., 2024a). Compared to our*post mortem*study on a single specimen, the effects of tractography errors may be mitigated in the*in vivo*group study because different sets of streamlines are affected in each individual. Further, the greater coverage of the V1–V2 interface*in vivo*compared to the*post mortem*slab means that streamlines had more space available to route around the problematic areas, even if they did not result in completely accurate trajectories. The 4 times higher spatial resolution*post mortem*may facilitate finer topographic mapping; nevertheless, this issue cannot be currently resolved given the lack of sufficient data and models.

The retinotopic organisation principle expects the SAF to connect all retinotopically corresponding points on the V1 and V2 cortex in both the eccentricity and polar angle directions. However, because of a lack of sufficient data and tractography models only a subset of the SAF in the polar angle direction could be mapped, confined to near the gyral crowns. This observation can be explained by the presence of the dominant long-range fibre pathways, including the thick and highly myelinated optic radiation tract penetrating V1, that prevent the SAF from being detected along the gyral walls in V1 and V2. If these SAF could be detected, an extension of the SAF sheet would be expected along the cortical normal direction at the sulcus, corresponding to the SAF connecting the entire cortical representation in the polar angle direction.

## 5 Conclusion

We mapped the detailed anatomy of SAF in a sub-region of the human V1–V2 cortex*in vivo*. The dense band of SAF connecting the V1 and V2 gyrus was detected in all participants. The SAF shapes were consistent with the cortical folding geometry, which was characterised mainly by a wide gyral angle across the group. Importantly, the SAF were organised into sheets with retinotopic topography, as demonstrated by retinotopic maps*in vivo*. Our findings were confirmed by a high-fidelity*post mortem*DWI tractography reference.

Our multi-modal MRI experimental setup*in vivo*established a direct link between the structure of SAF in the superficial white matter and functional organisation in the V1–V2 cortex. It demonstrated a SAF sheet structure that conformed to retinotopic topography as expected by the retinotopic organisation principle. The approach holds great potential for extending the study of brain anatomical and functional organisation to SAF as well as potential clinical applications, for example, to study cortical and SAF reorganisation in response to pathology and in neuronavigation in neurosurgery.

## Data and Code Availability

All data needed to evaluate the conclusions in the paper are present in the paper and/or the Supplementary Materials. Additional data and code related to this paper may be requested from the authors. The non-anonymised*in vivo*MRI data cannot be made publicly available, due to Ethics and Data Protection (European GDPR regulation) constraints. Processed and anonymised data can be provided on request.

## Author Contributions

Conceptualisation: F.M.A., E.K., and N.W. Methodology: F.M.A.; E.K., C.S. for*post mortem*sample, K.J.P., L.J.E. Software: F.M.A., D.C., K.J.P., and D.H. for fMRI. Formal analysis: F.M.A., D.H. for fMRI, E.K. and C.S. for*post mortem*DWI. Investigation: F.M.A., D.H. for fMRI, E.K., L.J.E., T.S., A.P., and J.P. for*post mortem*sample. Resources: N.W., M.M., and F.M.A. Data curation: F.M.A.; E.K., K.J.P., C.S., C.J., K.R., and T.N. for*post mortem*sample. Validation: F.M.A. Visualisation: F.M.A., L.J.E., and C.J. Writing—original draft: F.M.A. Writing—review and editing: all co-authors. Supervision: E.K., N.W., and T.N. for*post mortem*, RT for fMRI Project administration: F.M.A. Funding acquisition: N.W., E.K., S.M., and M.M.

## Funding

The*post mortem*tissue sample was provided by the former Brain Banking Centre Leipzig of the German Brain-Net, operated by the Paul Flechsig Institute, Center of Neuropathology and Brain Research, Medical Faculty, University of Leipzig. The authors would like to thank Enrico Reimer for providing the python script that created the retinotopic colourmaps for visualisation of SAF topography. The research leading to these results has received funding from the following sources: European Research Council under the European Union’s Seventh Framework Programme (FP7/2007-2013)/ERC grant agreement no 616905. BMBF (01EW1711A and 01EW1711B) in the framework of ERA-NET NEURON (N.W., S.M.). European Union’s Horizon 2020 research and innovation programme under the grant agreement No 681094 (N.W.). German Research Foundation priority program SPP2041 ‘Computational Connectomics’, project no 347592254 (KI 1337/2-2, WE 5046/4-2, MO 2397/5-2, MO 2249/3-2) (E.K., N.W., S.M., and M.M.). DFG Emmy Noether program MO 2397/4-1 and MO 2397/4-2 (SM). ERC (Acronym: MRStain, Grant agreement ID: 101089218, DOI: 10.3030/101089218). Views and opinions expressed are, however, those of the authors only and do not necessarily reflect those of the European Union or the European Research Council Executive Agency. Neither the European Union nor the granting authority can be held responsible for them (S.M.).

## Declaration of Competing Interest

The Max Planck Institute for Human Cognitive and Brain Sciences and Wellcome Centre for Human Neuroimaging have institutional research agreements with Siemens Healthcare. N.W. holds a patent on acquisition of MRI data during spoiler gradients (US 10,401,453 B2). N.W. was a speaker at an event organised by Siemens Healthcare and was reimbursed for the travel expenses.

## Supplementary Materials

Supplementary material for this article is available with the online version here:https://doi.org/10.1162/imag_a_00498.

## Supplementary Material

Supplementary Material

Supplementary Video 1

Supplementary Video 2
