# Supplementary material for: Short association fibres form topographic sheets in the human V1–V2 processing stream
Source: Imaging Neurosci (Camb). 2025 Mar 10;3:imag_a_00498. doi: 10.1162/imag_a_00498 (PMC12319882; doi:10.1162/imag_a_00498)
Supplement: Supplementary Material [file imag_a_00498-supp.pdf]

Supplementary materials

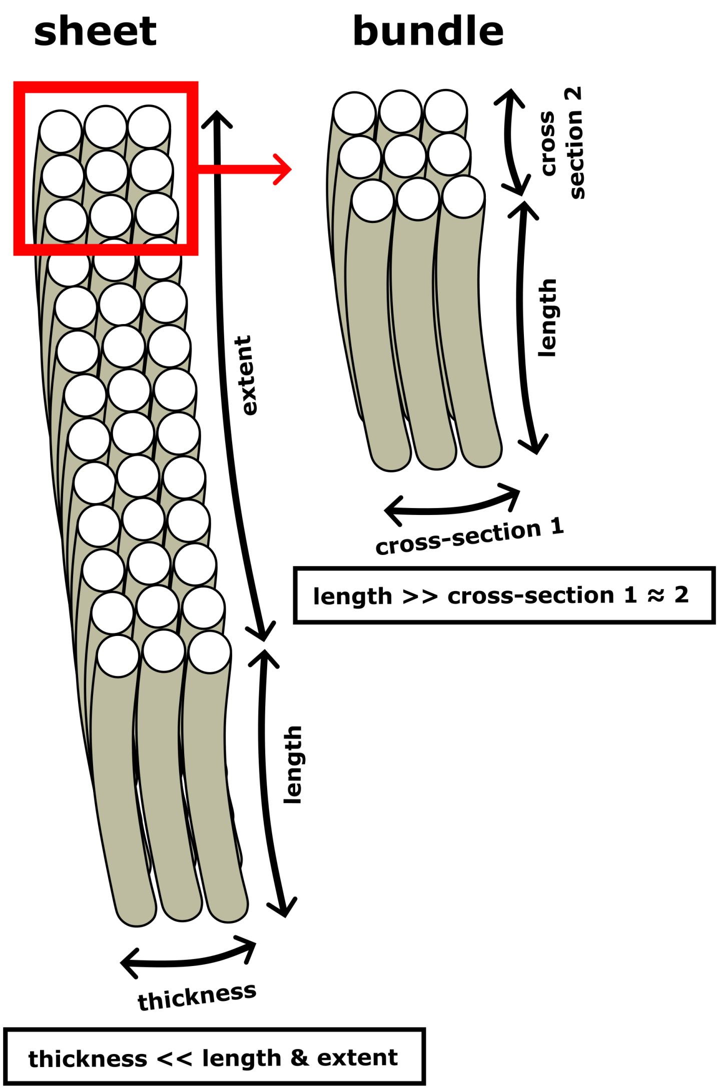

Fig. S1: Schematic representation of sheet and bundle. For the fibres to resemble a sheet, the thickness of the fibre layer must be much smaller than the length and the extent of the fibres. A bundle can be defined as a unit element of the sheet, where the length of the fibres is much larger than the thickness and extent, which make up the cross-section of the fibre bundle.

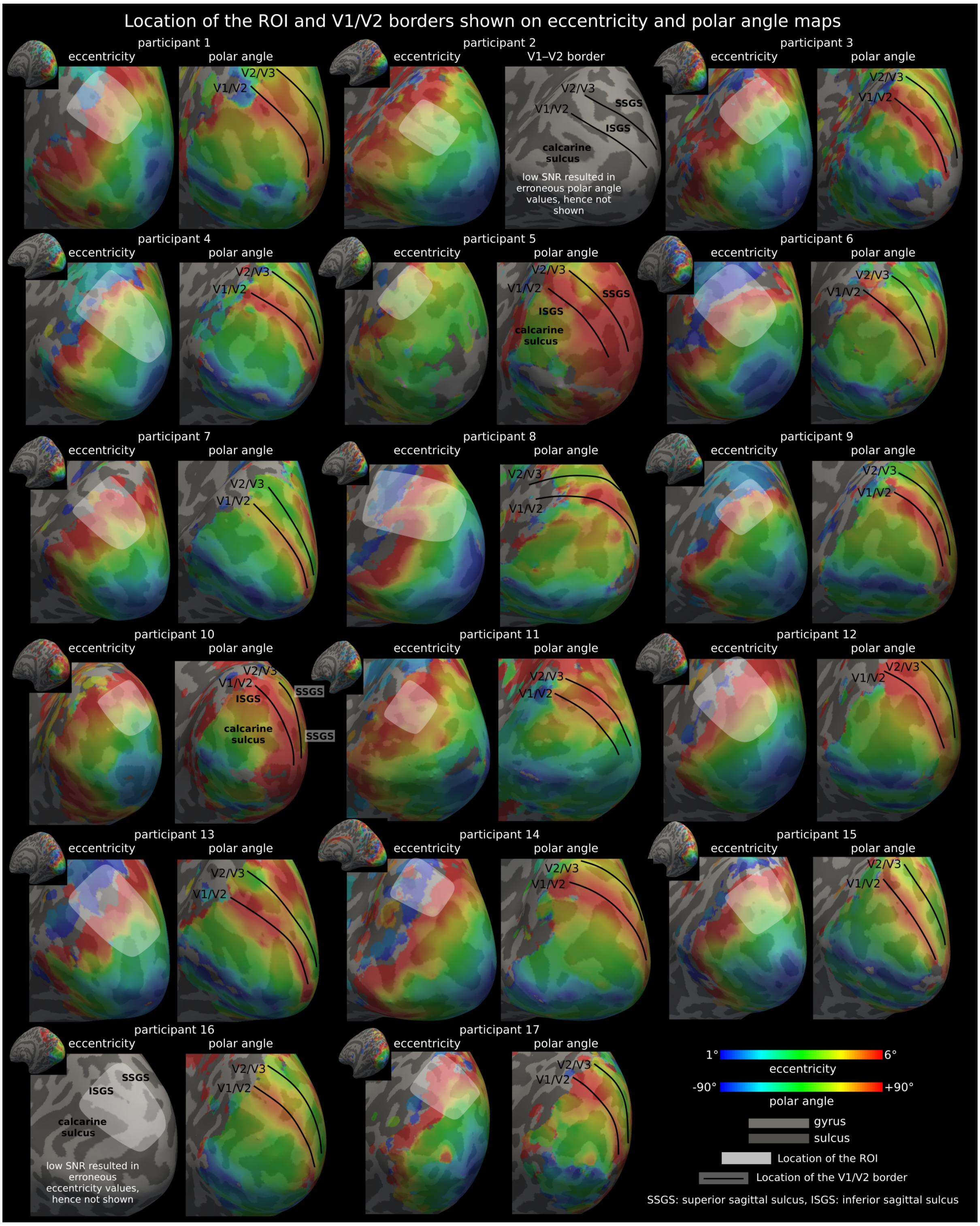

Fig. S2: Smoothed eccentricity and polar angle maps shown on inflated cortical surfaces for all participants. The approximate locations of the region of interest (ROI) are shown on the eccentricity maps, determined based on the appearance of the ISGS (a shallow sulcus) on the crown of the localised V1–V2 gyrus. The lower eccentricity border of the ROI was placed slightly posterior to the first appearance of ISGS, corresponding to approximately 3° in the eccentricity direction on average, estimated over the group. The delineated V1–V2 and V2–V3 borders are shown on the polar angle maps. Where SNR was low, giving erroneous functional maps (e.g. participants 2, 5 and 10) we used the sulcal landmarks to localise the V1 and V2 borders. ISGS and SSGS corresponded roughly to the locations of the V1–V2 and V2–V3 borders, the former running almost on top of the V1–V2 gyrus (See Fig. S5).

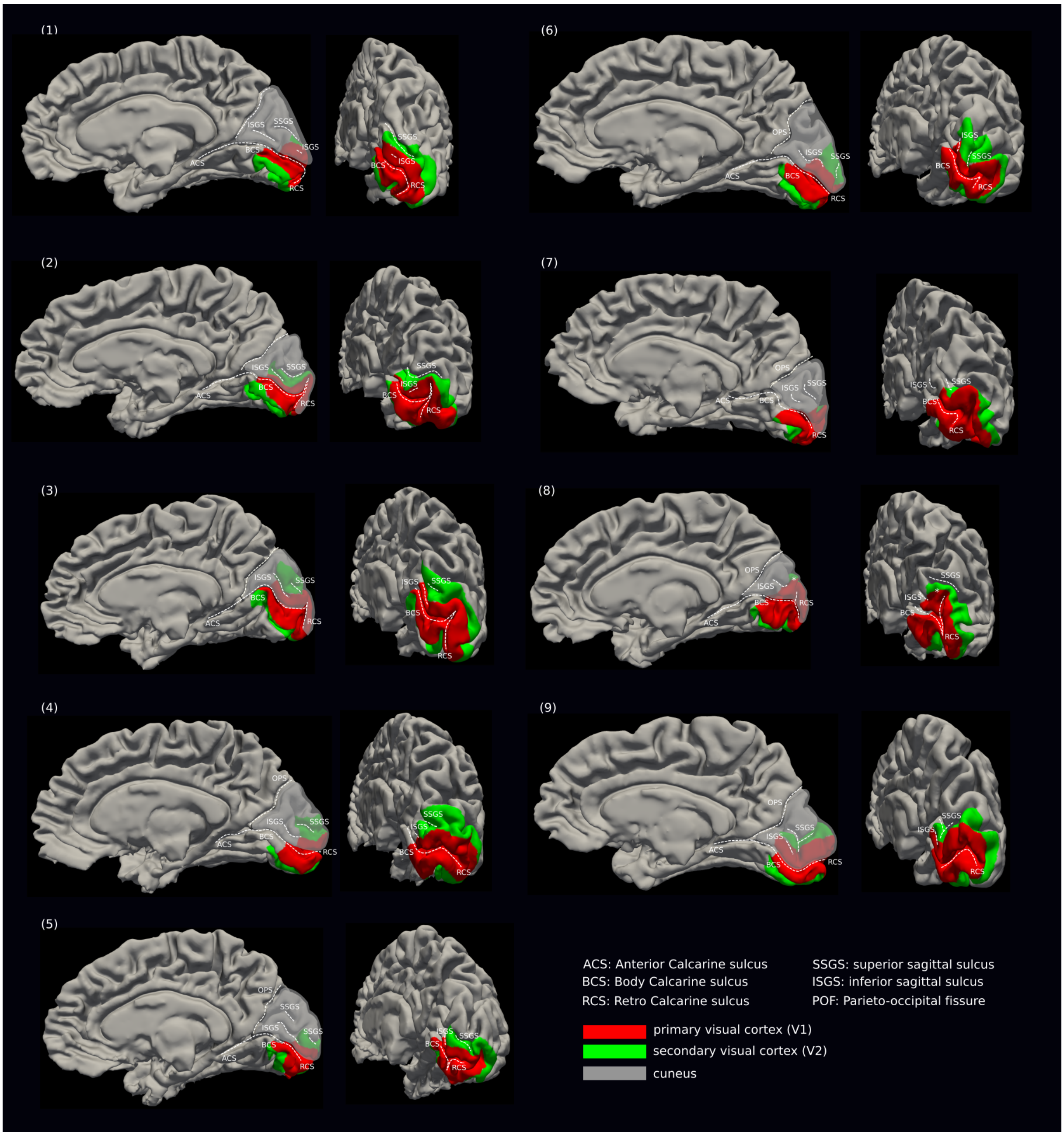

Fig. S3: Localisation of the V1–V2 gyrus within the ROI in the dorsal hemisphere (participants 1–9). Anatomical and functional landmarks are shown on sagittal and near coronal planes on the mid-cortical depth surfaces for each participant. The V1–V2 cortex was located in cuneus in the occipital lobe (highlighted area in sagittal view). The cuneus was delineated using the POF and the calcarine sulcus (CS). The sub-components of CS are shown (ACS, BCS and RCS). We localised the V1–V2 gyrus using the CS, ISGS and SSGS. ISGS and SSGS localisation were straightforward for most participants but less so for those with more ambiguous ISGS and/or SSGS according to guidelines in Iaria and Petrides, 2007 and/or with respect to V1 and V2 maps (e.g. V2 for participant 2 ends approximately at the location of the SSGS whereas for participant 3, it extends beyond the delineated SSGS). Non-contiguous portions of ISGS (participant 1) and SSGS (participant 5) were also observed.

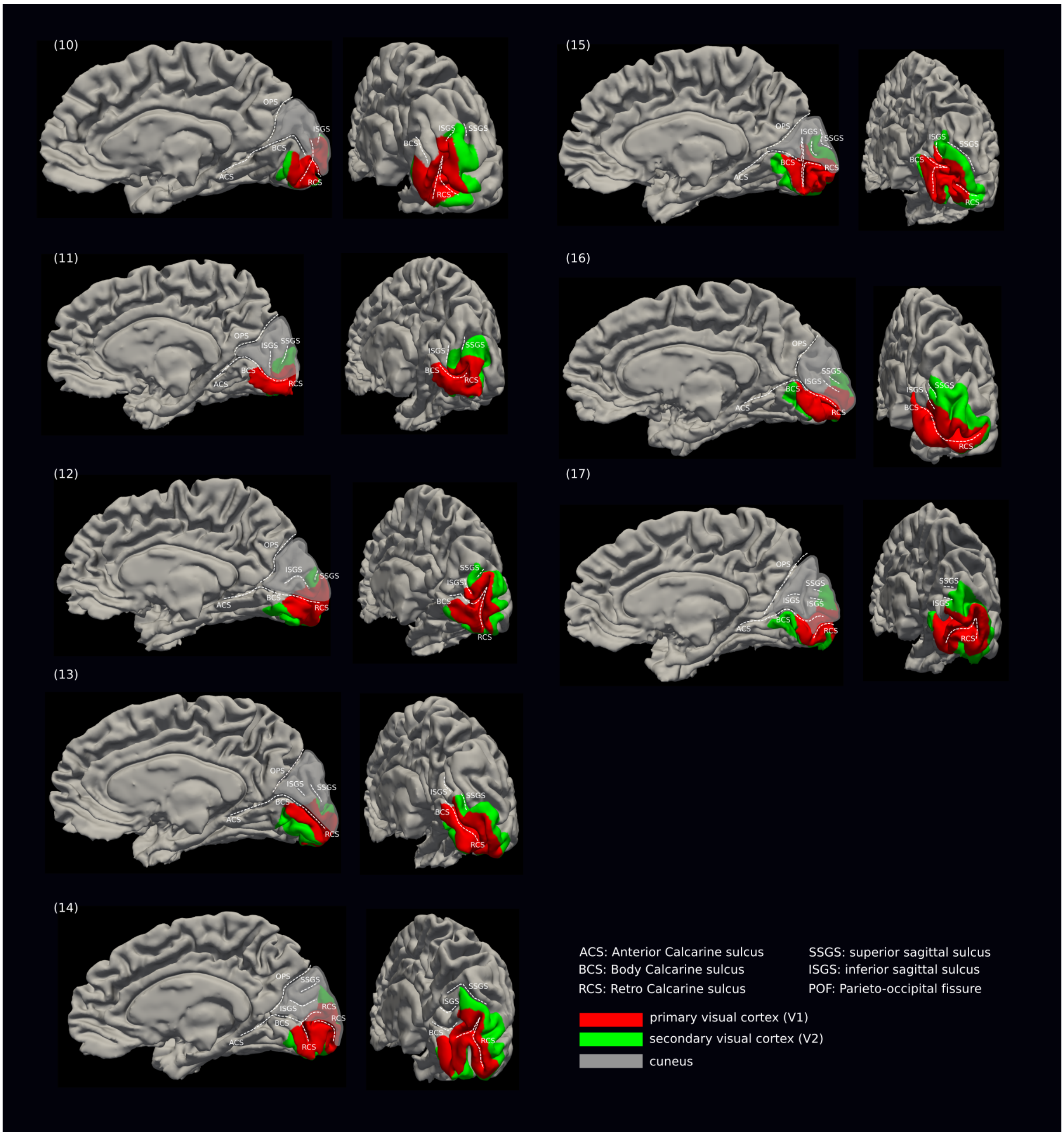

Fig. S4: Localisation of the V1–V2 gyrus within the ROI in the dorsal hemisphere (participants 10–17). Anatomical and functional landmarks are shown on sagittal and near coronal planes on the mid-cortical depth surfaces for each participant. The V1–V2 gyrus was located in cuneus in the occipital lobe (highlighted area in sagittal view). The cuneus was delineated using the POF and the calcarine sulcus (CS). The sub-components of CS are shown (ACS, BCS and RCS). We localised the V1–V2 gyrus using the CS, ISGS and SSGS.

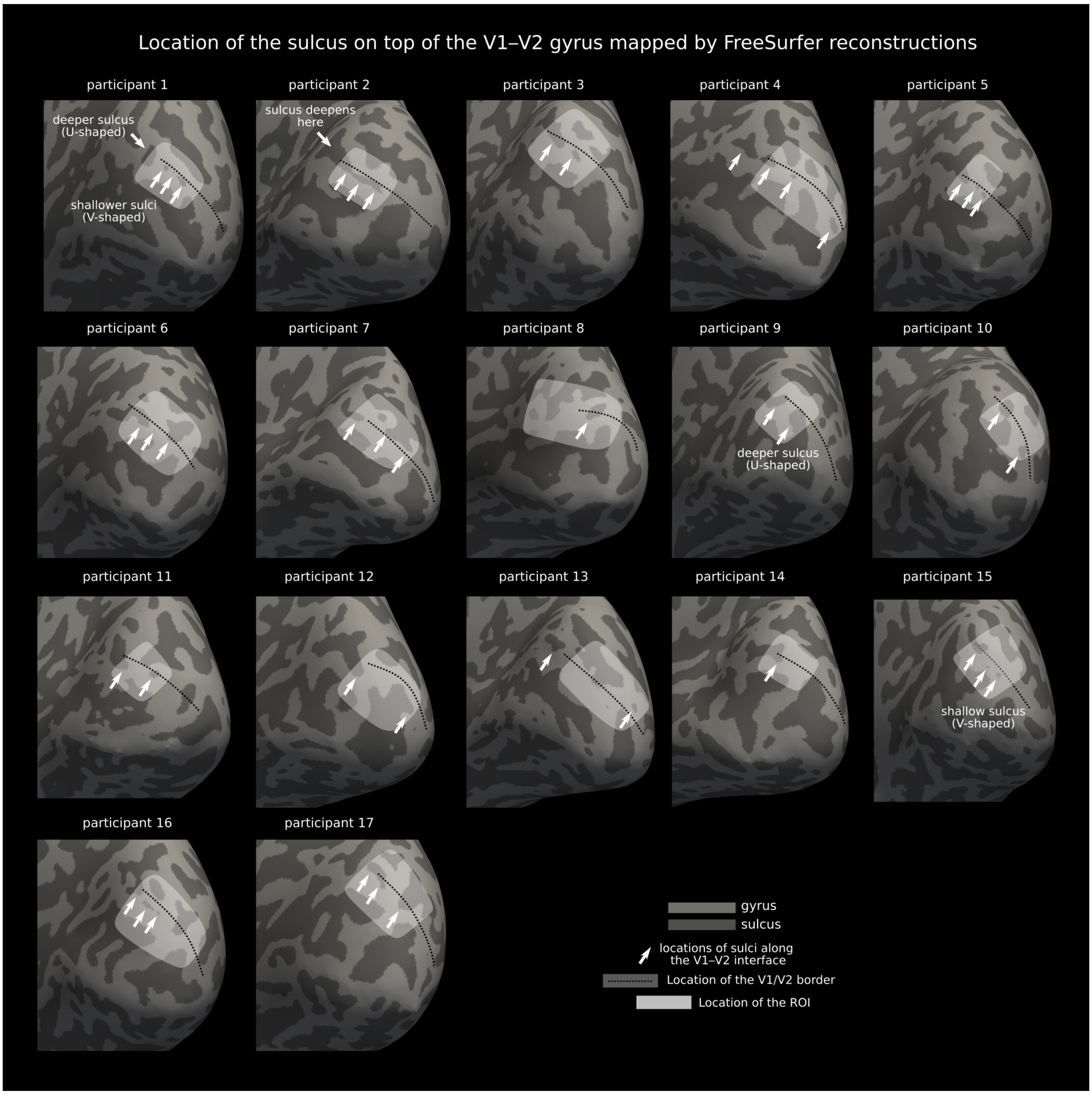

Fig. S5: Localisation of the shallow sulcus (ISGS) on the crown of the V1–V2 gyrus in all participants. The sulcus could be identified within the ROI (highlighted region) in all individuals on reconstructed curvature maps (threshold at 0 curvature) shown here on inflated cortical surfaces. The sizes, shapes and spatial distributions of the sulcus (white arrows) varied in the ROI across individuals and within each individual along the eccentricity direction. Both deep and shallow sulci were observed (see example for participant 1). For participants 1, 4, 16 and especially 2 the mapped sulcus was almost continuous in the eccentricity direction. Because the curvature of the sulcus was likely small relative to the 0.7 mm isotropic MP2RAGE spatial resolution it is possible that FreeSurfer reconstructions could not map it at all locations along the V1–V2 gyrus. This possibly explains the non-flat bending angles of SAF (mean $\pm$ standard deviation $152 \pm 15°$) mapped in locations where the sulcus could not be detected (i.e. flat curvature). Higher resolution MRI is required to further investigate the existence and appearance of the sulcus, as well as its relationship with SAF geometry.

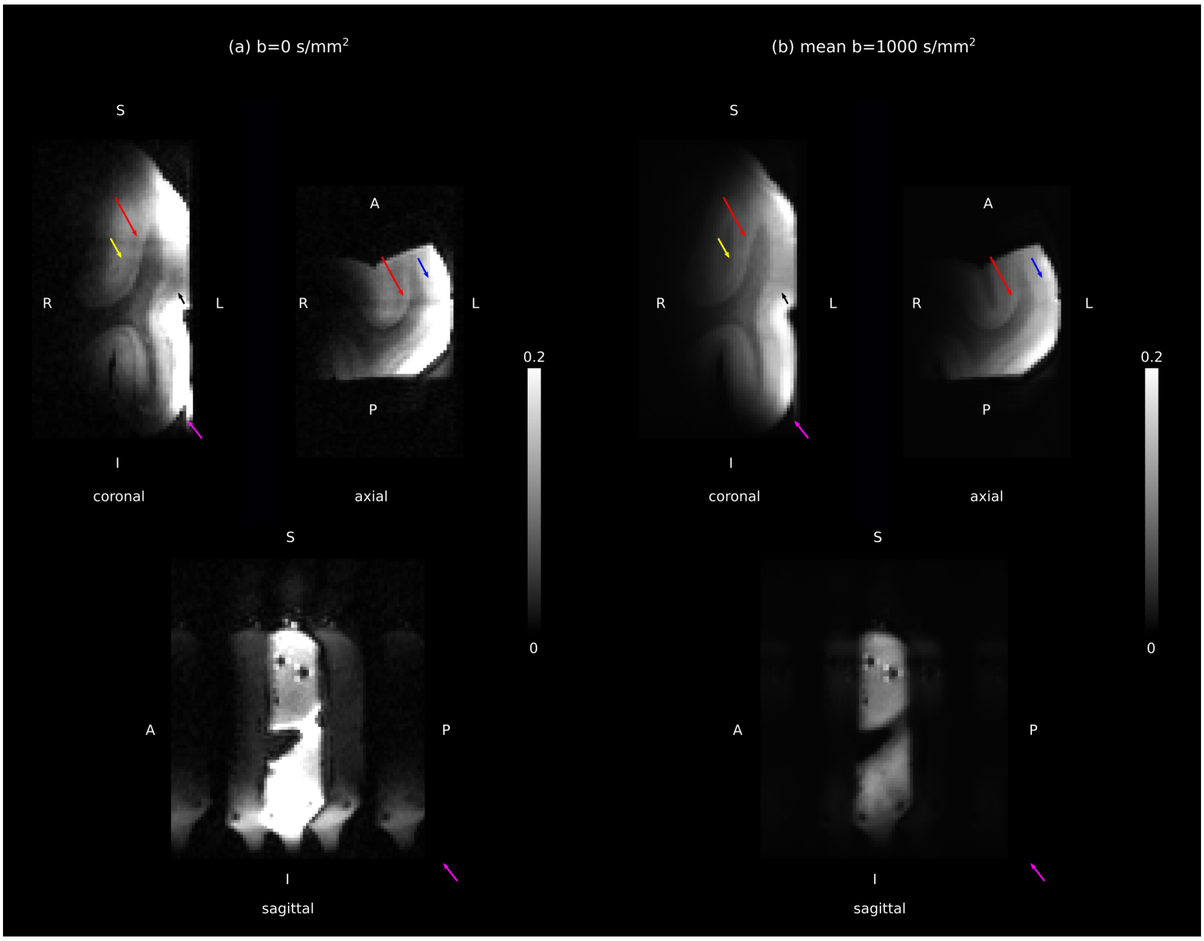

Fig. S6: The $b = 0$ s/mm^2 images *post mortem* were affected by localised artifacts and hence removed from the analysis. (a) A representative $b = 0$ s/mm^2 image from the DWI series showing the artifact on coronal, axial and sagittal slices in regions indicated by the arrows. (b) Mean $b = 1000$ s/mm^2 image computed based on the entire DWI series shows negligible diffusion weighting and significantly reduced artifacts compared to $b = 0$ s/mm^2. We used this image as a "pseudo b=0" image in the analysis. Arrows of the same colour point to the same location on all views. The ghosting artifact shown on the bottom panel in (a) and (b) would not have affected our results in the superficial white matter because it was located far from it. A: anterior, P: posterior, I: inferior, S: superior, L: left, R: right

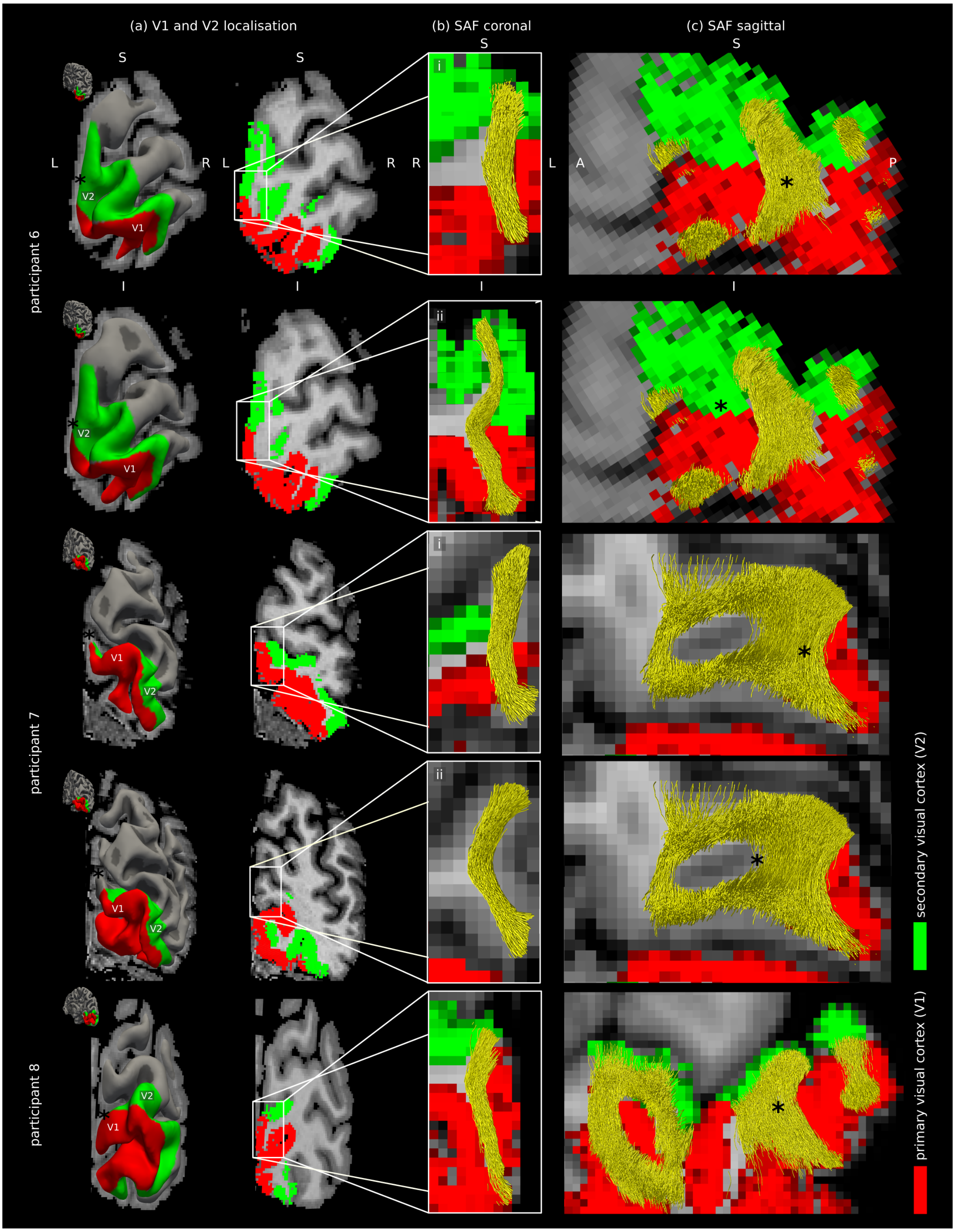

Fig. S7: The geometry of the SAF mapped in the localised V1–V2 ROI *in vivo* shown here for participants 6–8 (rows) was consistent across participants, characterised by a large bending angle. Results for participants 1–5 are shown in Fig. 3. (a) Surface (left) and volume (right) views of a representative coronal plane along the gyrus, looking from the posterior end of the brain. In diffusion space, (b) the mirror view of SAF is shown looking from the anterior end of the brain, corresponding roughly to the location of the plane in (a) marked by the asterisk (*) in (a,c), and (c) SAF mapped throughout the gyrus, pointing to a sheet geometry. The corresponding fibre distribution maps and sulcal landmarks are shown in supplementary Fig. S10. A: anterior, P: posterior, I: inferior, S: superior, L: left, R: right.

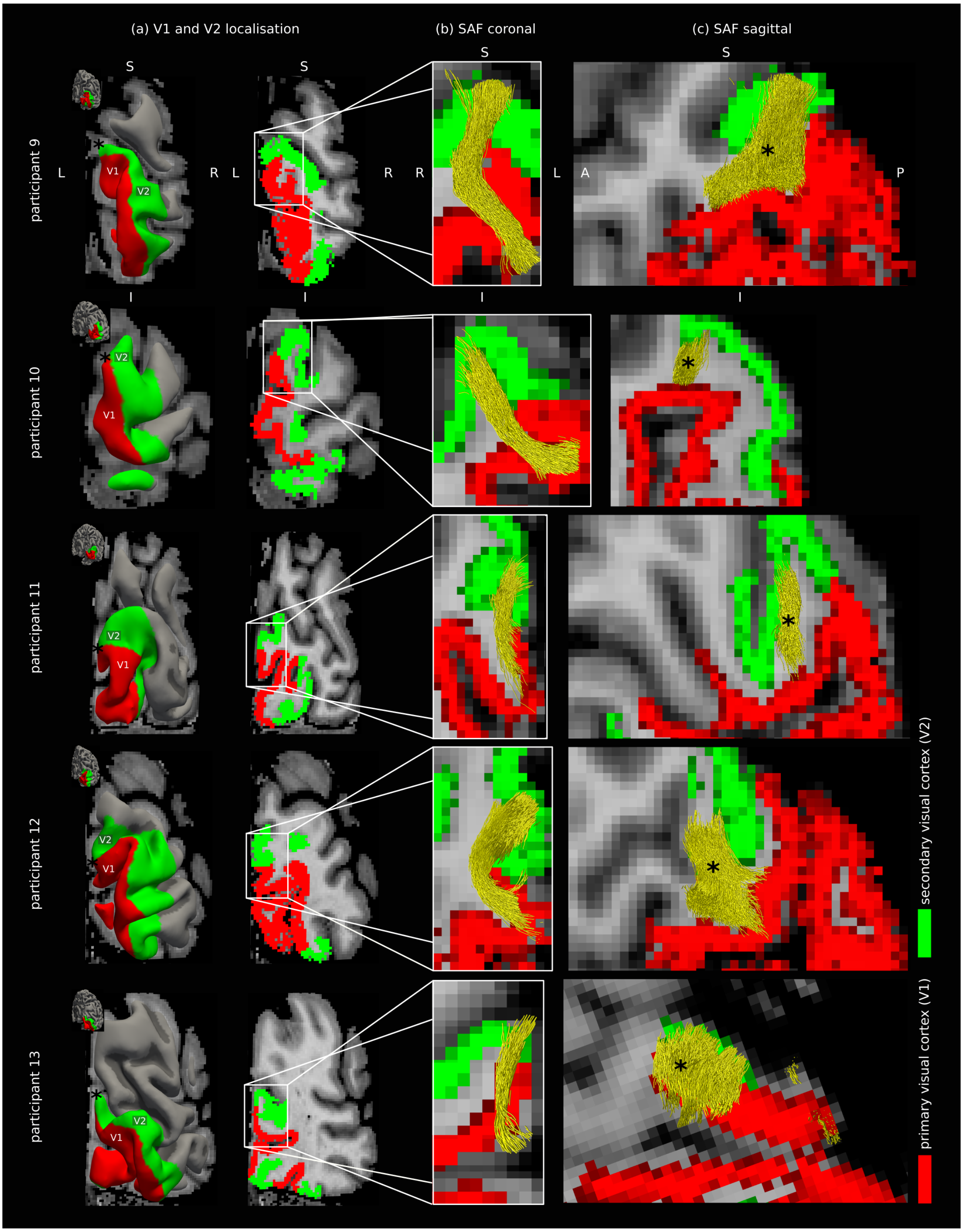

Fig. S8: The geometry of the SAF mapped in the localised V1–V2 ROI *in vivo* shown here for participants 9–13 (rows) was consistent across participants, characterised by a large bending angle. (a) Surface (left) and volume (right) views of a representative coronal plane along the gyrus, looking from the posterior end of the brain. In diffusion space, (b) the mirror view of SAF is shown looking from the anterior end of the brain, corresponding roughly to the location of the plane in (a) marked by the asterisk (*) in (a,c), and (c) SAF mapped throughout the gyrus, pointing to a sheet geometry. The corresponding fibre distribution maps and sulcal landmarks are shown in supplementary Fig. S10. A: anterior, P: posterior, I: inferior, S: superior, L: left, R: right.

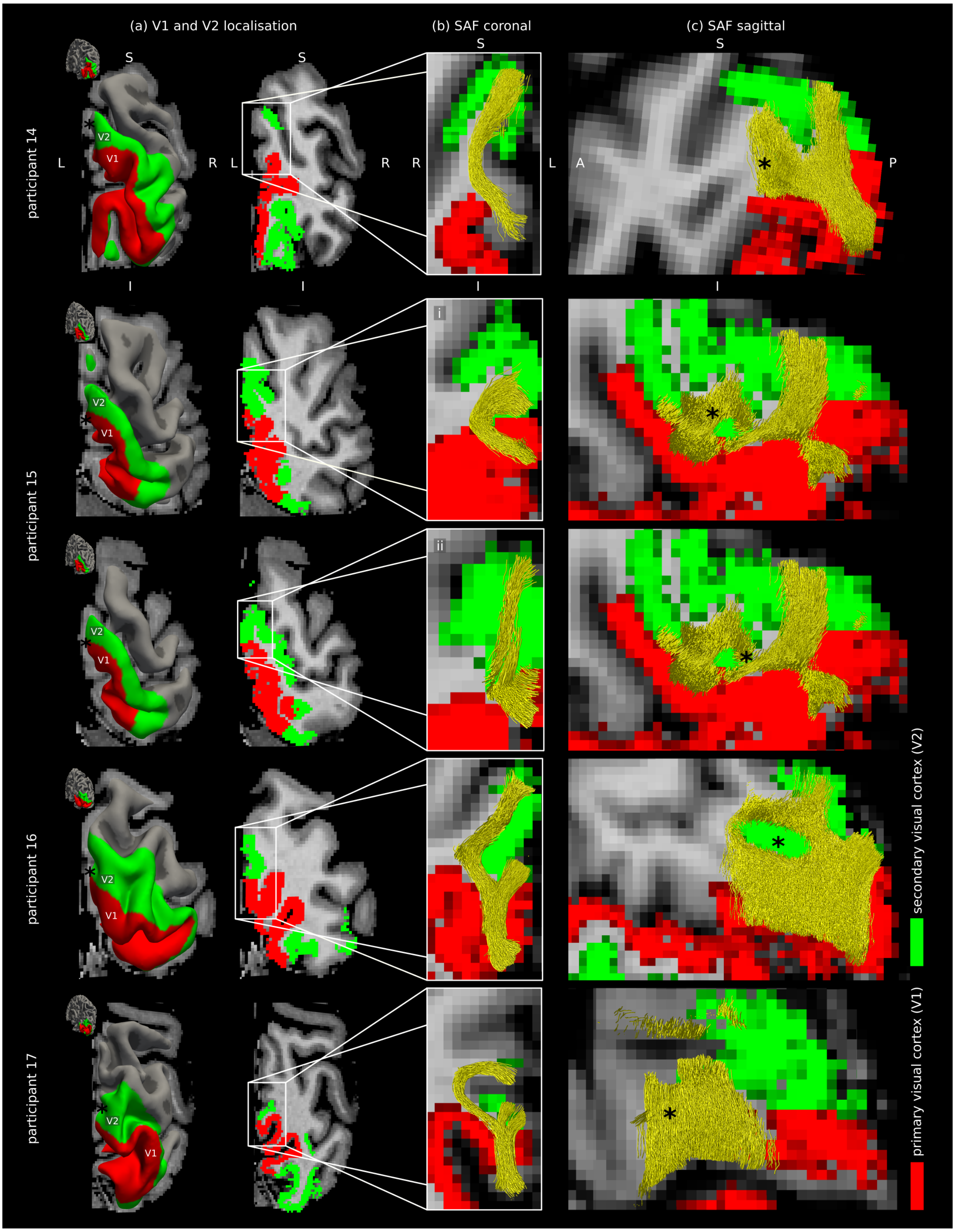

Fig. S9: The geometry of the SAF mapped in the localised V1–V2 ROI *in vivo* shown here for participants 14–17 (rows) showed some variability. For participant 15 in slice (i), the SAF were strongly U-shaped and for participants 16 and 17, they comprised a longer and a shorter segment. (a) Surface (left) and volume (right) views of a representative coronal plane along the gyrus, looking from the posterior end of the brain. In diffusion space, (b) the mirror view of SAF is shown looking from the anterior end of the brain, corresponding roughly to the location of the plane in (a) marked by the asterisk (*) in (a,c), and (c) SAF mapped throughout the gyrus, pointing to a sheet geometry. The corresponding fibre distribution maps and sulcal landmarks are shown in supplementary Fig. S10. A: anterior, P: posterior, I: inferior, S: superior, L: left, R: right.

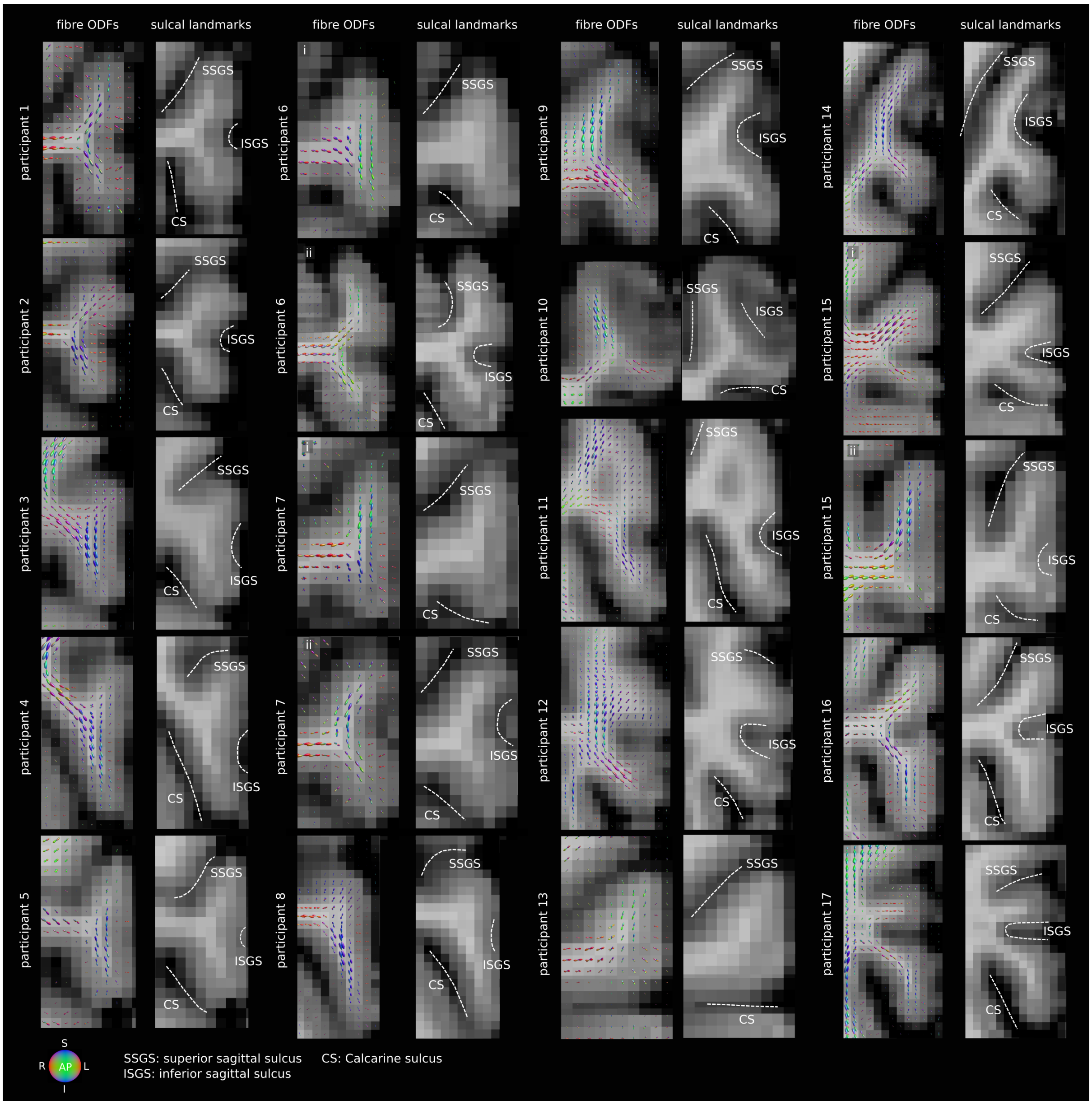

Fig. S10: Fibre distributions and sulcal landmarks in the explored V1–V2 gyrus for all participants *in vivo*. The shown coronal planes correspond to the locations of the planes in Figs. 3b, S7b, S8b and S9b. The fibre ODF maps show small or no crossing peaks in the superficial white matter for all participants, facilitating precise SAF tractography. The localised sulcal landmarks enable precise identification of the V1–V2 gyrus *in vivo*. The ODF colour coding defined by the reference sphere indicates the orientation of the mapped fibres. A: anterior, P: posterior, I: inferior, S: superior, L: left, R: right.

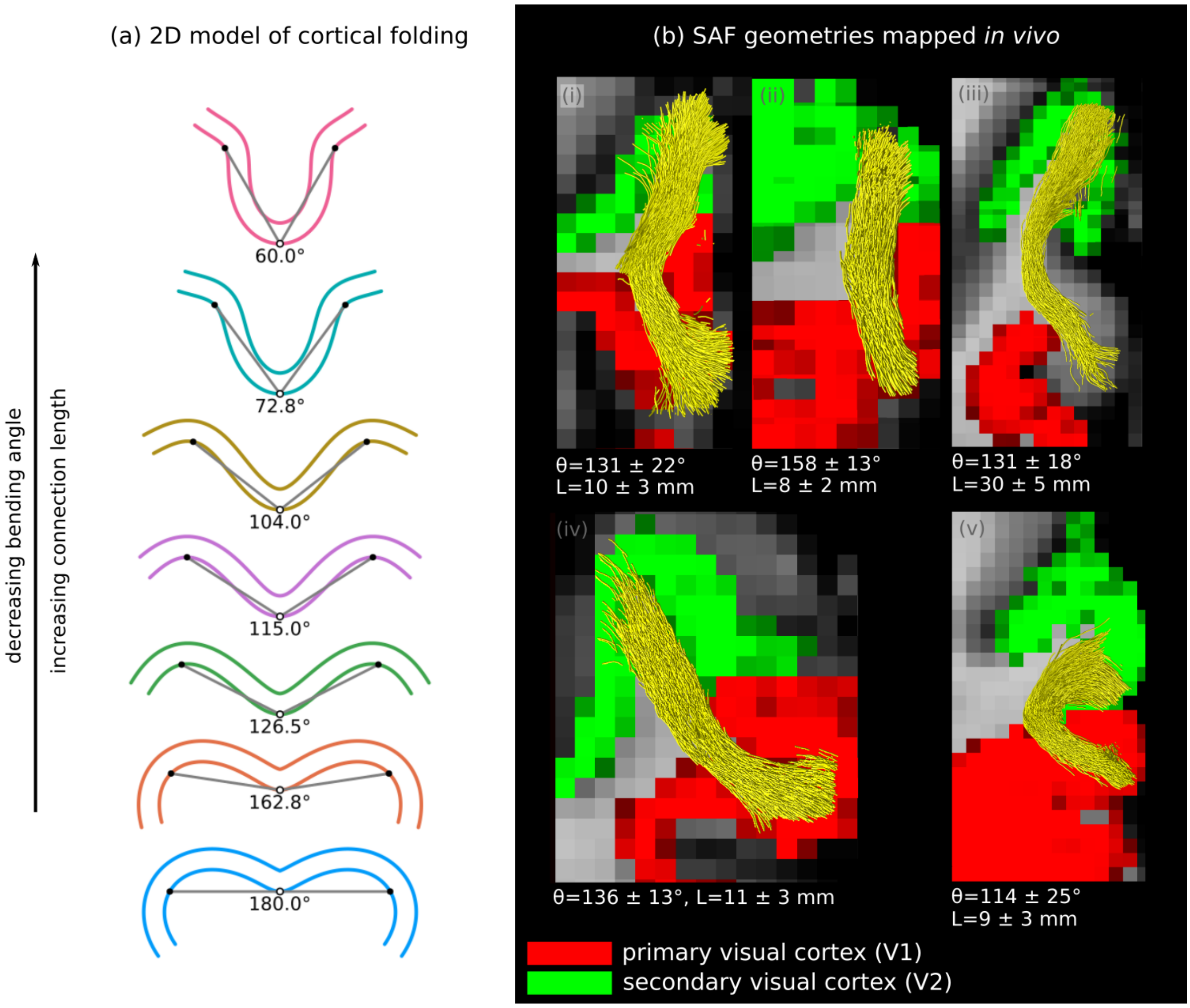

Fig. S11: Characterising SAF shapes using a length and a bending angle metric. (a) A 2D model of cortical gyrification shows how the geometry of the fibre pathways connecting the two peaks on the circle can change as a function of peak locations. The lengths of the grey lines connecting the gyral crowns (black dots) and sulcal fundi (white dots) are equal in each case. The closer the crowns are, the relatively longer the shortest path connecting them becomes, as it has to deviate from the straight path connecting the crowns and the fundus and go around the walls of the sulcus. This also means that the shape of the pathway changes and becomes more U-shaped as opposed to the V- (and straight) shapes of the relatively shorter pathways. SAF geometries are thus strongly linked to the cortical folding patterns. To characterise their geometries, we defined a bending angle computed as the angle enclosed by the two vectors connecting the two streamline endpoints (schematically represented here by the gyral crowns) to the point of maximum curvature along the streamline (schematically represented here by the sulcal fundus). Larger angles are closer to V-shapes and smaller angles to U-shapes. Previous *in vivo* studies point to the functional importance of the different SAF shapes (Chauvel et al., 2022; Rajimehr & Tootell, 2009). (b) V1–V2 SAF geometries mapped *in vivo* showed some variation in geometry, however, most resembled V-shapes (i-iv) while only a few resembled U-shapes (v). The examples shown here are taken from representative participants. Although setting a threshold is not trivial, angles greater than approximately 110° appeared to be characteristic of V-shapes.

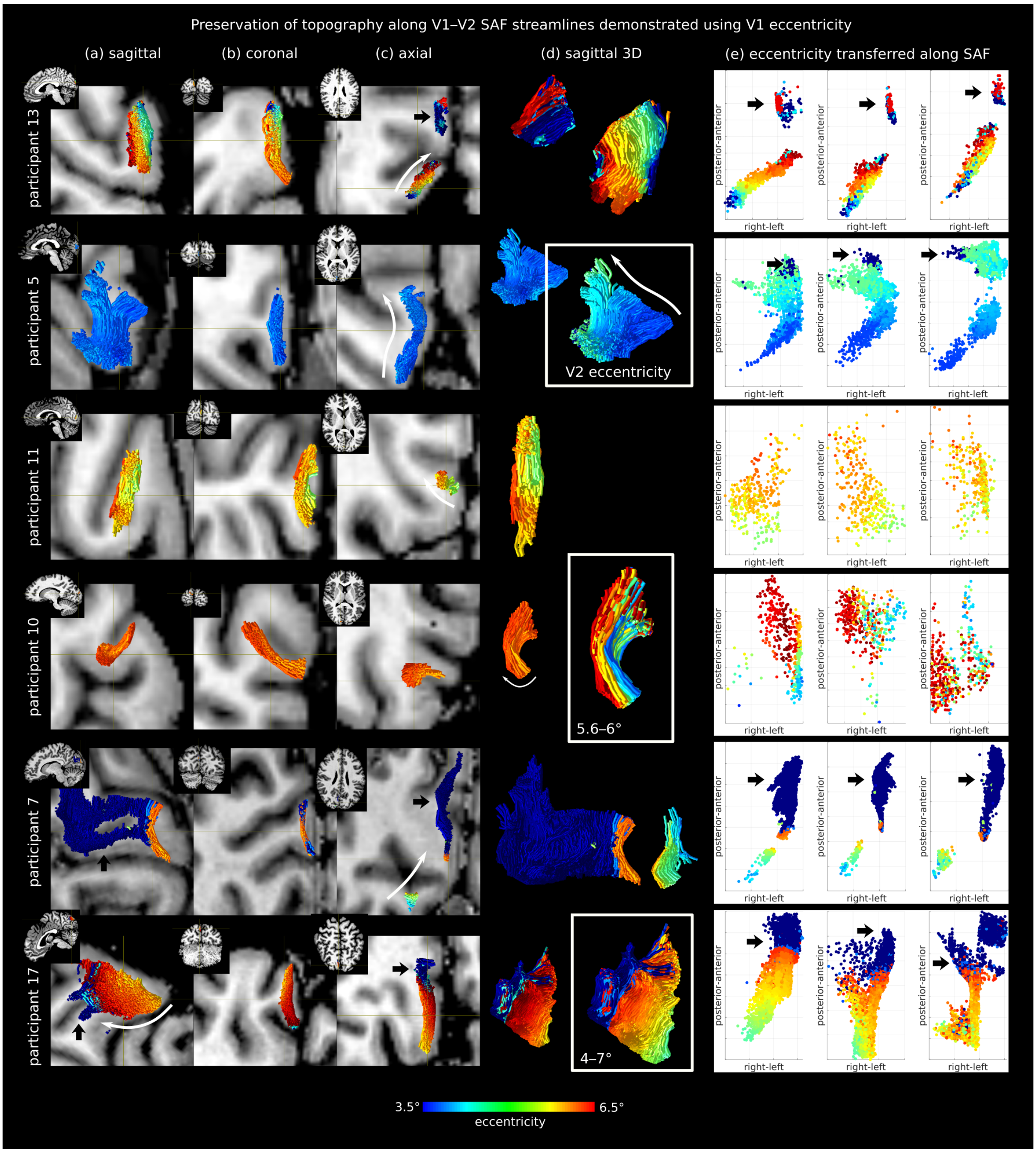

Fig. S12: Eccentricity mapped onto the V1–V2 SAF streamlines shows along-track topography *in vivo*. 6 out of 17 participants are shown. Eccentricity at each location on the V1 cortex was projected onto its corresponding streamline shown here on (a) sagittal, (b) coronal and (c) axial slices, and (d) a 3D sagittal view. The relative locations of the SAF in the eccentricity direction are deducible by the colour scale, and white arrows show the direction of increasing eccentricity. (e) Along-track topography is also shown by mapping eccentricity onto the first, middle and last coordinates along each SAF streamline. To better appreciate the topography, V2 eccentricity is shown instead of V1 eccentricity in (d,e) for participant 5 and the colour scale is adjusted to the shown ranges for participants 10 and 17. Recall that to enable SAF mapping throughout a larger area of the cortex covering also regions beyond the functionally mapped V1 and V2 cortex, we manually extended the V1 and V2 borders along the sulcal landmarks where these could be visually identified on MP2RAGE reconstructed curvature maps. For participants 13 and 17, the most anterior located streamlines corresponded to beyond the functionally mapped cortex, indicated by black arrows. For participant 7, a greater portion of the streamlines corresponded to this region. The colour scale does not apply to these regions and may be ignored.

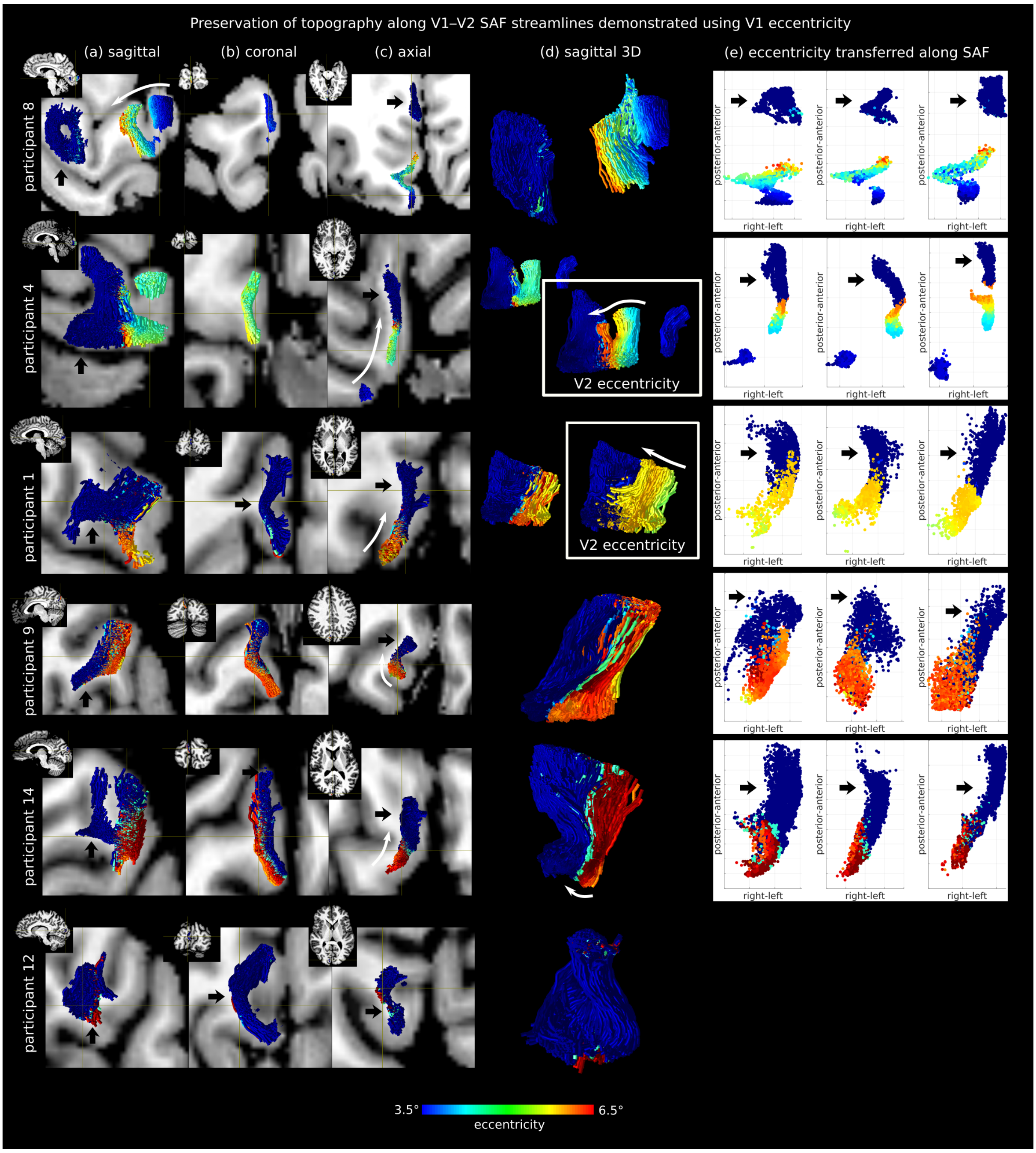

Fig. S13: Eccentricity mapped onto V1–V2 SAF streamlines shows along-track topography *in vivo*. 6 out of 17 participants are shown. Eccentricity at each location on the V1 cortex was projected onto its corresponding streamline shown on (a) sagittal, (b) coronal and (c) axial slices, and (d) a 3D sagittal view. The relative locations of the SAF in the eccentricity direction are deducible by the colour scale, and white arrows show the direction of increasing eccentricity. (e) Along-track topography is also shown by mapping eccentricity onto the first, middle and last coordinates along each SAF streamline. To better appreciate the topography, V2 eccentricity is shown instead of V1 eccentricity in (d,e) for participants 1 and 4. For the 6 participants shown here, the mapped SAF corresponded partially (or entirely, for participant 12) to beyond the functionally mapped V1 and V2 cortex, indicated by black arrows (see also Fig. S12 caption). Therefore, along-track topography (e) is not shown for participant 12. Participant 16 is also not shown because low SNR of the eccentricity map resulted in erroneous eccentricity values (see supplementary Fig. S5). The colour scale does not apply to these regions and may be ignored.

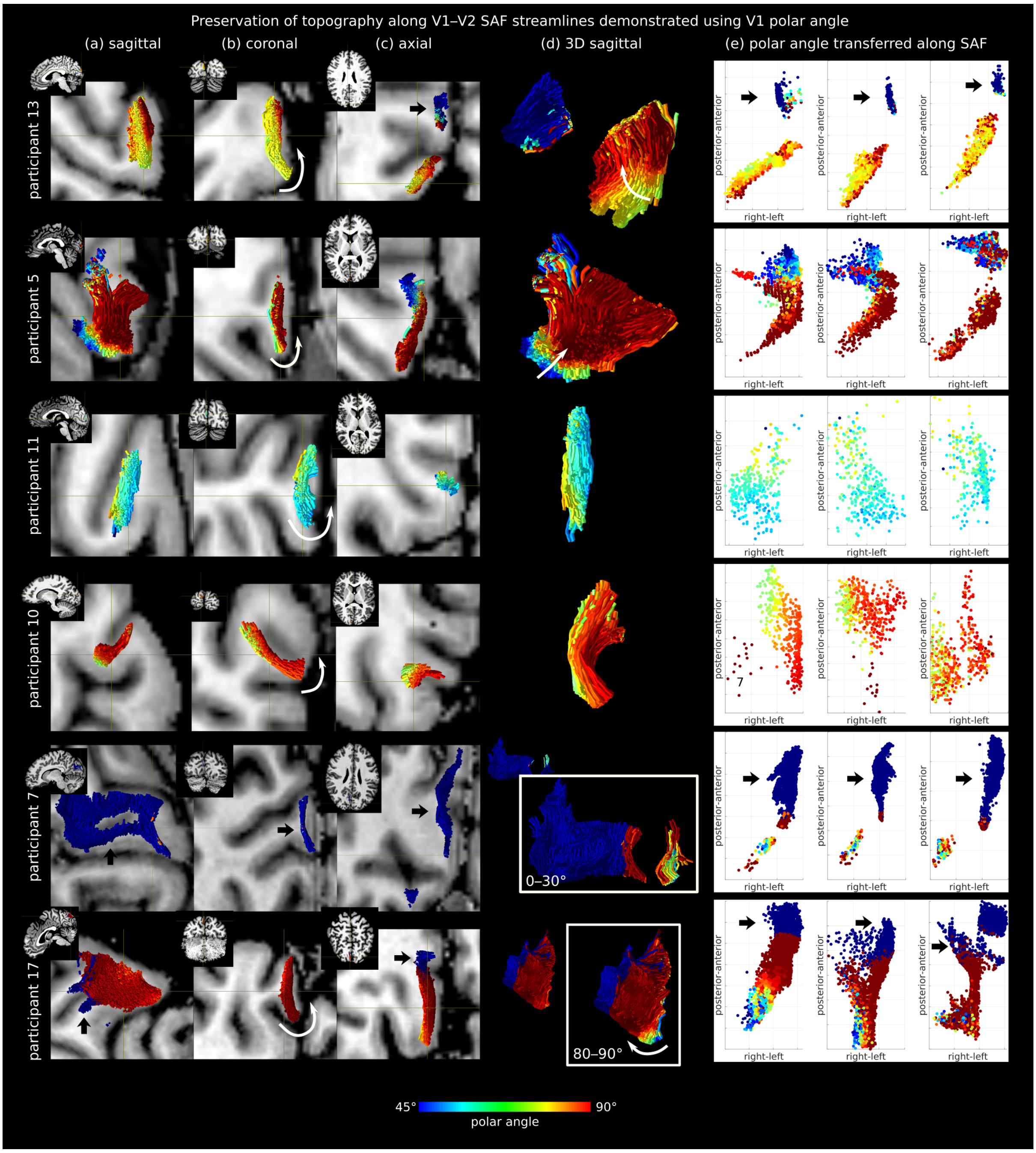

Fig. S14: Polar angle mapped onto V1–V2 SAF streamlines shows along-track topography *in vivo*, however, to a lesser extent compared to eccentricity. 6 out of 17 participants are shown. Polar angle at each location on the V1 cortex was projected onto its corresponding streamline shown on (a) sagittal, (b) coronal and (c) axial slices, and (d) a 3D sagittal view. The relative coverage in the polar angle direction by SAF is deducible by the colour scale, and white arrows show the direction of increasing polar angle. (e) Along-track topography is also shown by mapping polar angle onto the first, middle and last coordinates along each SAF streamline. To better appreciate the topography, the colour scale is adjusted to the shown ranges for participants 7 and 17 in (d,e). For participants 13 and 17, the most anterior located streamlines corresponded to beyond the functionally mapped cortex, indicated by black arrows (see also Fig. S12 caption). For participant 7, a greater portion of the streamlines corresponded to this region. The colour scale does not apply to these regions and may be ignored.

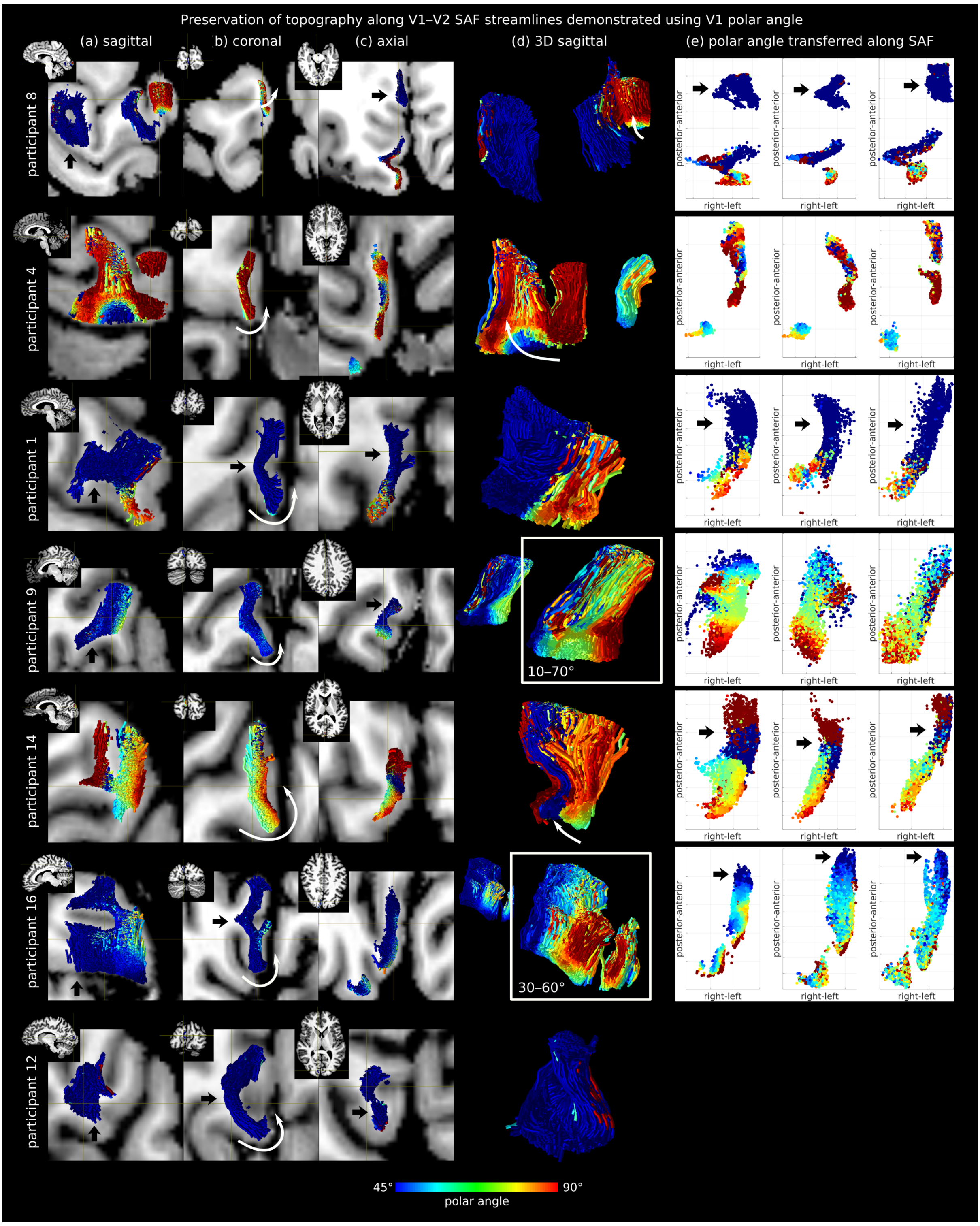

Fig. S15: Polar angle mapped onto V1–V2 SAF streamlines shows along-track topography *in vivo*, however, to a lesser extent compared to eccentricity. 7 out of 17 participants are shown. Polar angle at each location on the V1 cortex was projected onto its corresponding streamline shown on (a) sagittal, (b) coronal and (c) axial slices, and (d) a 3D sagittal view. The relative coverage in the polar angle direction by SAF is deducible by the colour scale, and white arrows show the direction of increasing polar angle. (e) Along-track topography is also shown by mapping polar angle onto the first, middle and last coordinates along each SAF streamline. To better appreciate the topography, the colour scale is adjusted to the shown ranges for participants 9 and 16 in (d,e). For the 6 participants shown here, the mapped SAF corresponded partially (or entirely, for participant 12) to beyond the functionally mapped cortex, indicated by black arrows. Therefore, along-track topography (e) is not shown for participant 12. The colour scale does not apply to these regions and may be ignored.

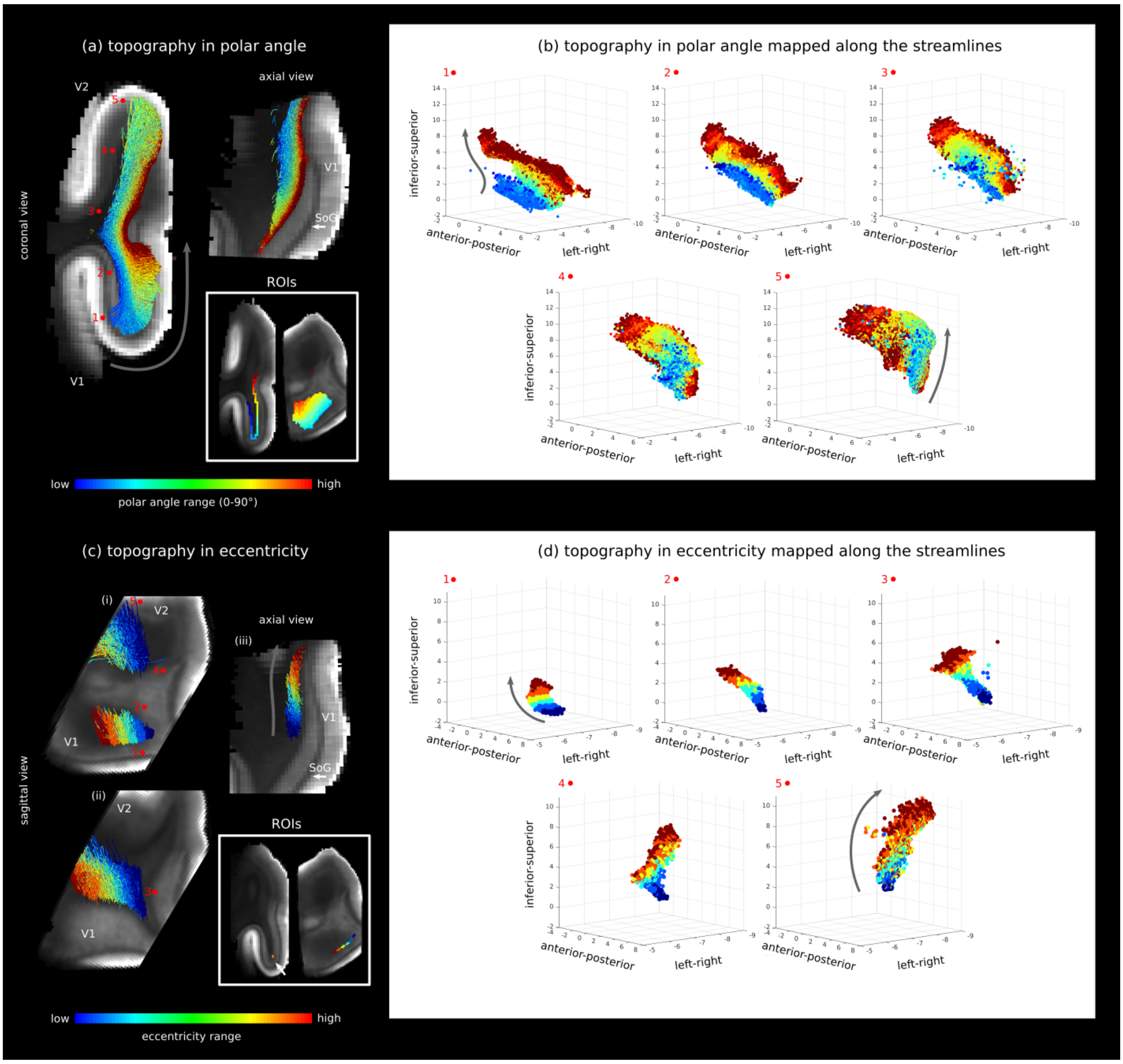

Fig. S16: Topography of the V1–V2 SAF mapped in the *post mortem* specimen in the (a,b) polar angle and (c,d) eccentricity directions. The SAF streamlines were clustered using two different sets of manually created labels in V1 in the expected polar angle and eccentricity directions, shown in the ROIs panel in (a) and (c). (a,b) For polar angle, topography was mapped at 5 points along the streamlines, showing it was less preserved closer to V2. The axial view in (a) corresponds to a plane between locations 2 and 3 in the coronal view. Arrows in (b) show the direction of increasing polar angle. (c,d) For eccentricity, topography was also mapped at 5 points along the streamlines, showing it was also slightly disrupted closer to V2. The axial view (c-iii) corresponds to the plane in location 3 in the sagittal view (c-ii). Arrows in (d) show the direction of increasing eccentricity. The eccentricity ROIs were created along the crown of the V1 gyrus, indicated by the arrow in the ROIs panel in (c). The V1-specific Stria of Gennari (SoG) is also indicated.

Caption to Video V.1. Eccentricity mapped in V1 cortex by fMRI projected along V1–V2 SAF streamlines for the three participants shown in Fig. 5, separately.

Caption to Video V.2. Polar angle mapped in V1 cortex by fMRI projected along V1–V2 SAF streamlines for the three participants shown in Fig. 6, separately.
